# Influence of the porosity on the photoresponse of a liquid crystal elastomer

**DOI:** 10.1098/rsos.150700

**Published:** 2016-04-06

**Authors:** Emre Kizilkan, Jan Strueben, Xin Jin, Clemens F. Schaber, Rainer Adelung, Anne Staubitz, Stanislav N. Gorb

**Affiliations:** 1 Department of Functional Morphology and Biomechanics, Zoological Institute, Kiel University, Am Botanischen Garten 1–9, 24118 Kiel, Germany; 2 Otto-Diels-Institute for Organic Chemistry, Kiel University, Otto-Hahn-Platz 4, 24118 Kiel, Germany; 3 Institute for Materials Science, Functional Nanomaterials, Kiel University, Kaiserstrasse 2, 24143 Kiel, Germany; 4 Institute for Organic and Analytical Chemistry, University of Bremen, Leobener Straße NW 2 C, 28359 Bremen, Germany

**Keywords:** photoresponsive materials, liquid crystal elastomers, azobenzene, porous polymers

## Abstract

Azobenzene containing liquid crystal elastomers (LCEs) are among of the most prominent photoresponsive polymers due to their fast and reversible response to different light stimuli. To bring new functions into the present framework, novel modifications in bulk material morphology are required. Therefore, we produced azobenzene LCE free-standing films with different porosities. While the porosity provided macroscopic morphological changes, at the same time, it induced modifications in alignment of liquid crystal azobenzene units in the films. We found that a high porosity increased the photoresponse of the LCE in terms of bending angle with high significance. Moreover, the porous LCE films showed similar bending forces to those of pore-free LCE films.

## 1. Introduction

Stimuli-responsive polymers that can be actuated by light or other stimuli have gained much attention in recent years [1,2]. Light-activated shape-memory and shape-changing polymers demonstrate reversible macroscopic shape changes through light-triggered molecular motion. One of the most prominent light-responsive polymers is liquid crystal elastomer (LCE) [3–5]. The principle of these LCEs was initially theoretically predicted by de Gennes [6] and they were first synthesized by Küpfer & Finkelmann [7]. In particular, LCEs with azobenzene moieties in their mesogens have been most widely used as photoresponsive polymers due to their fast response to different light wavelengths and their strong absorption [8,9]. By illumination with ultraviolet (UV) light with wavelengths of typically 350–370 nm, the *trans* isomers of azobenzene molecules switch to the *cis* isomers, thereby changing their geometry from a molecular length of 9 Å in the *trans* configuration to 5.5 Å in the *cis* configuration [10,11]. This enormous change is reversible and can be changed back to the stable *trans* configuration thermally or by illumination with light with wavelengths in the visible range [12,13]. Macroscopic motions caused by these reversible molecular size changes of azobenzene LCEs were demonstrated, such as robotic arm movement, twisting, high-frequency oscillation or switchable bending [9,11,14–16].

Implementing the LCEs into technological applications requires novel modifications in the material's properties in order to enhance the photoresponse or to bring new functions to the present framework. With convenient processability, lower density and bigger apparent surface area for external stimuli, porous polymers are in many cases more advantageous than solid materials [17]. Moreover, porous structure provides an easy incorporation of multiple functional materials with different properties in three-dimensional space. For example, recently, porous LCEs have been used as a three-dimensional scaffold for cell cultures [18].

In this work, we produced for the first time photoresponsive azobenzene LCE films with different porosities. The porous LCEs demonstrated improved photomechanical responses, which were evaluated by measuring their bending angles under illumination with either UV or visible light. The results indicated that higher porosities of the LCE films increased the final bending angle. We have also shown the force actuation of these porous LCEs during UV illumination and compared the results with pore-free LCE. Moreover, the photoswitchability of highly porous LCE was demonstrated in terms of bending forces generated during the photoisomerization of azobenzene, when the highly porous LCE samples were alternately illuminated by UV and visible (VIS) light sources. In search for the sources of the increased bending of the porous films, we tested whether this effect can be attributed to a change in the materials' effective elastic modulus. Furthermore, we examined the influence of the porosity to the alignment of the azobenzene mesogenes in pore-free films and porous films. We conclude that the modifications in the alignment of the mesogens and other morphological differences in the presence of porosity are factors for the enhanced mechanical properties of the porous LCE films.

## 2. Results and discussion

**Figure 1.**
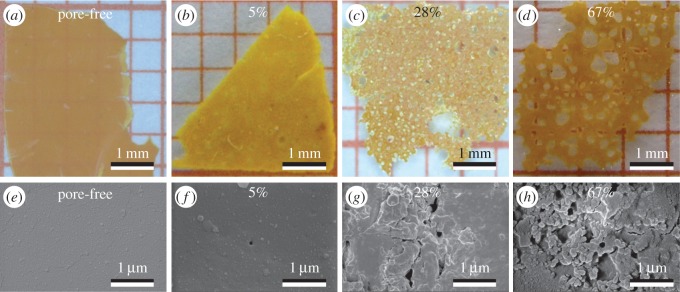
LCE films with different degree of porosity. (*a–d*) Photographic images enable to identify the degree of macro-porosity. (*e–h*) Small pores observed down to a size of approximately 50 nm by scanning electron microscopy (SEM) images.

Three different porous LCE films with air volumes of 5%, 28%, 67% and pore-free LCE films were produced. In the porous films, the pores had diameters of 0.05–100 µm for the film with 5% porosity, 0.05–400 µm for the film with 28% porosity and 0.05–500 µm for the film with 67% porosity (figure 1).

**Figure 2.**
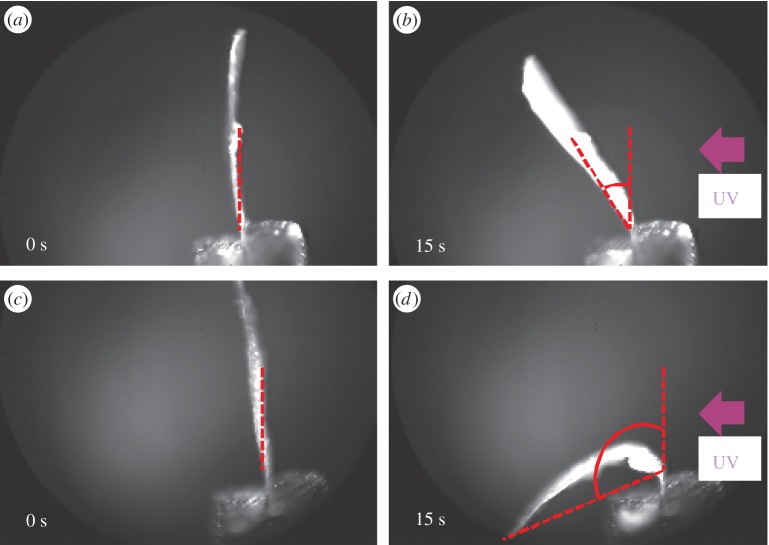
Bending of 5% (*a,b*) and 67% (*c,d*) porous LCE films (side view) at different points in time (0 and 15 s) under UV illumination. The bending angles are indicated by the dashed lines.

**Figure 3.**
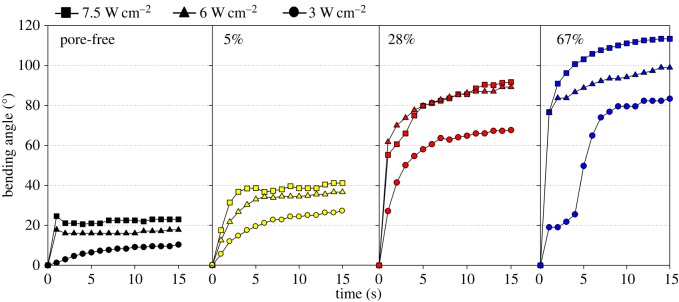
Time-dependent increasing bending angle of differently porous (pore-free, 5, 28 and 67%) LCE films under UV light illumination with light intensities of 7.5, 6 and 3 W cm^−2^.

The photoresponse of the LCE films was characterized by bending angle measurements during illumination of UV light (*λ* = 365 nm). As we used the same monomers, cross-linker and a similar polymerization process compared to the work reported previously, it can be assumed that in these cases the penetration depth of light is consistent with previous work on azobenzene LCEs [19]. The films were fixed at their one end, and the displacement angles were measured by comparing the position of other end before and after the UV light illumination. The light-triggered bending motions were recorded in a time-dependent sequence (figure 2). The samples of pore-free, 5, 28 and 67% porous LCE free-standing films were observed during the illumination with a UV light source for 15 s each, where the light intensities were adjusted to 7.5, 6 and 3 W cm^−2^ by varying the distance of the light source from the sample (see the electronic supplementary material). As the films did not revert to their initial position when the UV illumination was turned off, we concluded that the bending of the films was not thermally induced. All the bending angles reached a saturation plateau at 7 s illumination time due to the saturation of the amount of photoisomerized azobenzene units. Samples of porous LCEs with increasing porosity demonstrated higher bending angles in comparison with pore-free LCE at any intensity of UV light used (figure 3). The light intensity itself also had an influence on the bending behaviour. The higher the intensity, the larger were the final bending angles. The final bending angles for the films with 28 and 67% porosity were higher than those for the film with 5% porosity and pore-free films for any intensity used. In addition, the bending angles of the 5% porous LCE film exceeded those of the pore-free LCE film at any intensity of UV light illumination. The bending angle of the 67% porous sample under 3 W cm^−2^ intensity of UV light illumination was smaller than the bending angles of the 28% porous sample under 7.5 and 6 W cm^−2^ intensities.

As the light intensity decreased, the final bending angle also decreased. At 1–5 s of illumination, two samples of 67% porous films reached higher bending angles, and more quickly than the 5% porous and the pore-free films. Interestingly, at 2 s, the bending angles for the 5% porous and 28% films illuminated with 7.5, 6 and 3 W cm^−2^ were higher than those for the 67% porous films illuminated at 3 W cm^−2^. We speculate that this result may be due to the more irregular surface of the highly porous sample. Therefore, the more porous sample might demonstrate non-steady bending behaviour at the observation direction during photoisomerization of azobenzene units in the film.

**Figure 4.**
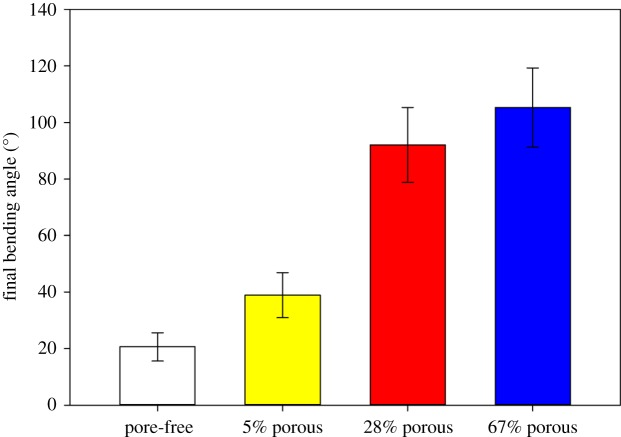
Mean final bending angles (± s.d.) of the differently porous LCE films after illumination with UV light for 15 s.

The average final bending angles (each based on testing of nine samples) obtained at the three different intensities of illumination were 20° for the pore-free film, 39° for the film with 5% porosity, 92° for the film with 28% porosity and 105° for the film with 67% porosity (figure 4). The differences in the final bending angles between all porous films and the pore-free samples, between the 5 and 28%, and the 5 and 67% porous films were statistically highly significant (*p* ≤ 0.001, one-way ANOVA, Tukey test). However, the final bending angle difference of 28 and 67% porous films was not statistically significant (*p* = 0.056, one-way ANOVA, Tukey test).

**Figure 5.**
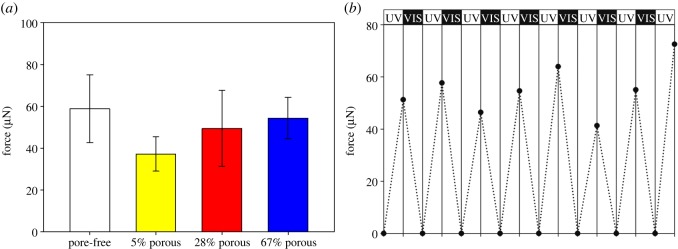
Force actuation of LCE films with different porosities. (*a*) Comparison of force actuation by LCE films during bending when illuminated by 7.5 W cm^−2^ light intensity for 15 s with pore-free, 5, 28 and 67% porosity. (*b*) Forces arising by consecutive photoswitching of an LCE film with 67% porosity by alternate illumination with UV and visible light for 15 s.

A further important aspect is how much mechanical force can be generated by this photoresponse. The light-triggered force actuation of the pore-free and the 5, 28 and 67% porous LCE films were measured with three samples each at an illumination of 7.5 W cm^−2^ light intensity by deflecting a glass cantilever force probe in contact with the free-standing LCE film. On average, the pore-free films exhibited higher forces than porous films (figure 5*a*). Among the porous LCE films, a higher degree of porosity resulted in higher bending forces. In total, 28 and 67% porous films showed forces statistically not significantly different from the pore-free LCE (pore-free versus 28% porous: *p* = 0.242; pore-free versus 67% porous films: *p* = 0.236; one-way ANOVA, Tukey test). However, the difference between the pore-free and the 5% porous LCE is statistically highly significant (*p* = 0.004, one-way ANOVA, Tukey test).

In order to demonstrate the photoswitchability of the porous LCE films, a sample of a 67% porous film was exposed to cycles of consecutive illumination by UV (*λ* = 365 nm) and visible light (VIS, *λ* = 455 nm) with intensities of 7.5 W cm^−2^ resulting in reversible back and forth bending motions (figure 5*b*).

The mechanical behaviour of these porous films can be modelled by the bending of a plate structure. In mechanics, the force required to bend a plate structure to a unit curvature is proportional to $EH^{3}/\;12(1-\upsilon^{2}),$ where *H* is the thickness of the plate, *E* Young's modulus and *υ* Poisson's ratio [20]. If equal bending forces are applied to films with the same thickness and apparent area, more porous films will bend more easily because the overall effective elastic modulus (*E*′) decreases. The origin of the forces, which lead to bending of the film in this case, is the light-triggered isomerization of the azobenzene mesogens. While there are fewer azobenzene mesogens in a more highly porous volume when compared with solid bulk material, the bending forces might be expected to be reduced for this film with its less total volume. Thus, we can first assume that the bending force generated is proportional to the volume of azobenzene mesogens. With this assumption, it would be expected to observe similar bending angles for high and low porous films. However, on the contrary, the experimentally determined bending angles were higher with higher porosity. Therefore, the bending angle difference of these differently porous LCEs must have a different cause. It may be explained by the different ratios between photoisomerized and non-photoisomerized volumes in high and low porous LCE films.

**Figure 6.**
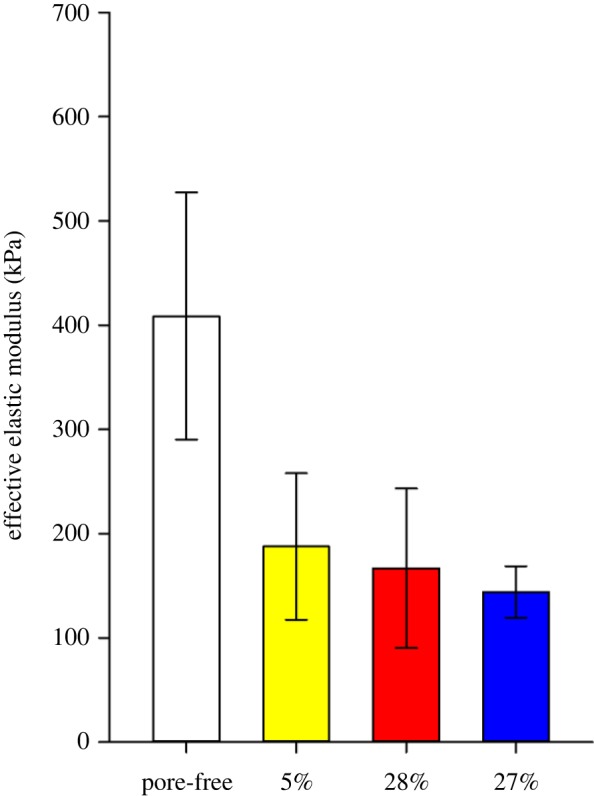
Effective elastic modulus (*E*′) of pore-free, 5, 28 and 67% porous LCE films. *E*′ of the pore-free film is statistically highly significantly larger than that of the porous films (*p* ≤ 0.001, one-way ANOVA, Tukey test), number of tests *n* = 20 each). Among porous films, the differences in *E*′ are not statistically significant (5 versus 28% porous: *p* = 0.342; 5 versus 67% porous films: *p* = 0.133; 28 versus 67% porous films: *p* = 0.672; one-way ANOVA, Tukey test).

When comparing the values of the effective elastic modulus values of the LCEs, *E'*of the pore-free film is significantly higher than those of the porous films (figure 6). However, the small difference in *E'* between 5, 28 and 67% porosity cannot explain the highly significant differences between the final bending angles of the UV-illuminated samples. Thus, we propose another explanation.

Because the LCE films bent away from the direction of the UV light source, they can be assumed to have mesogens with a perpendicular alignment directory to the film surface [12,21]. Under UV light illumination, such aligned mesogens of the azobenzene LCE films expand their length at the surface of the illuminated area due to the *trans*–*cis* photoisomerization of the azobenzene structural motifs. This leads to an orientation disorder of the domain structure on the surface layers and induces an expansion of the volume which absorbed UV light. As there is no UV-induced volume change in the deeper layers, the LCE film bends away from the light source.

**Figure 7.**
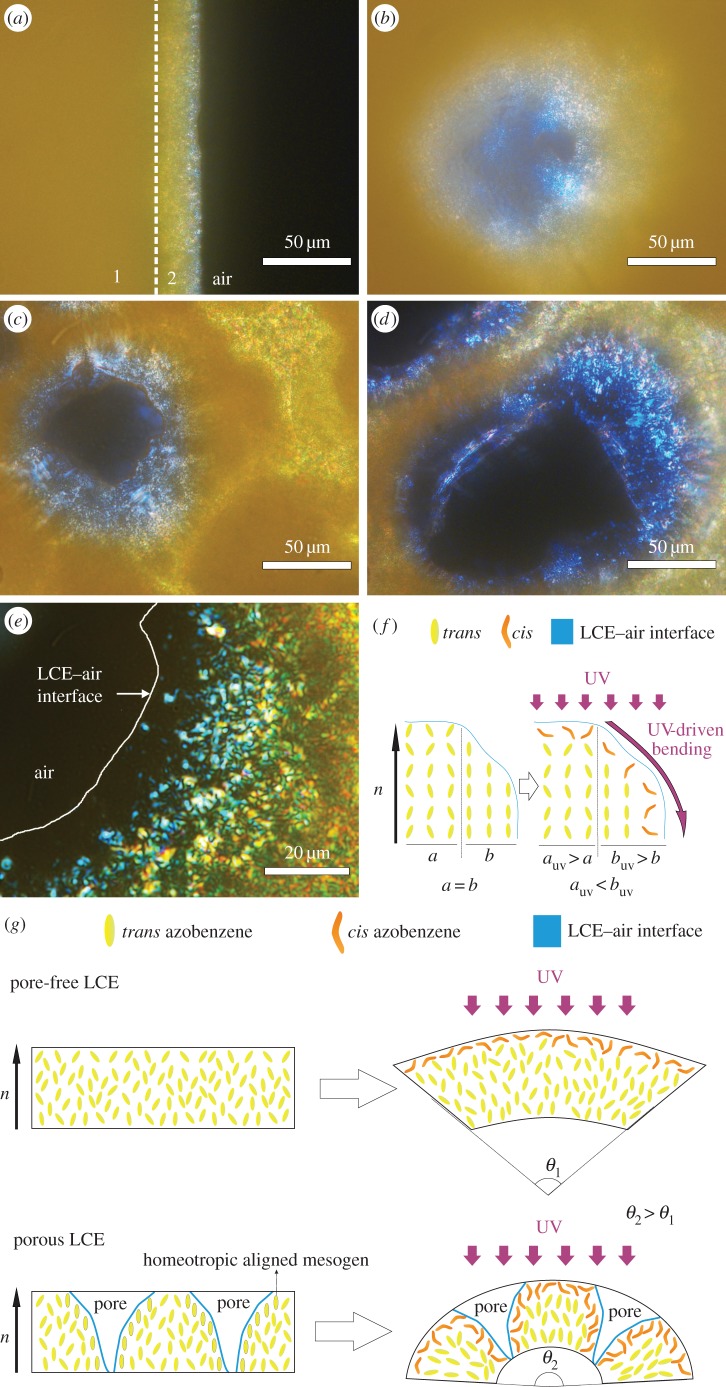
(*a*) Pore-free (area 1) was cut at an angle of 45° relative to surface to see through LC phase alignment (area 2). The area 1 appears like isotropic solid surface, which is probably caused by the increased thickness. (*b*) The 5% porous, (*c*) the 28% porous and (*d*) the 67% porous LCE films. (*e*) Closer look with higher magnification POM image to the LCE–air interface of a pore in a 67% porous film. The black area at the left is air space of a pore. Homeotropically aligned areas of the LCE mesogens close to the LCE–air interface (right of the white line) remain black. The different colours (whitish for nematic LC alignments close to the LCE–air interface, orange as the thickness increases) are due to different polarization angles of the transmitted polarized light, passing through nematic LC. (*f*) The area interface is separated to two parts; *a* = nematic alignment and *b* = homeotropic alignment at the border. If the surface of LCE is illuminated with UV light, the area illuminated expands. The homeotropically aligned area expands more than nematic area since the expansion at the *y*-axis is bigger for completely perpendicularly aligned azobenzene mesogenes during photoisomerization. The alignment directory of mesogens (*n*) is parallel to film surface. (*g*) Schematic of bending of pore-free (upper line) and porous (lower line) LCE films by UV light illumination. Note that the porosity provides a relatively bigger illumination area and enables the absorption of the UV light deep into the film. Thus, for porous films the proportion of photoisomerized volume to non-photoisomerized volume is bigger compared with pore-free films with the same thicknesses.

A contribution to the increased bending angles of porous films may be the mesogen local alignment. With the presence of pores, liquid crystal (LC) mesogen alignment differs from pore-free LCEs. Polarized optical microscopy (POM) revealed a polydomain nematic orientation of the liquid crystalline phase (figure 7*a*). In order to demonstrate the dependence of thickness on appearance of LC phases in POM images, the pore-free film was cut 45° to the surface (figure 7*a*). The surface seems like an isotropic solid surface (figure 7*a*-1); however, throughout the area cut until the LCE–air interface, birefringences of nematic LC alignments can be observed via crossed polarizers (figure *7a*-2). In the porous films, with the introduction of pores onto the surface, local thickness differences and modifications of the LC phase alignment are observed (figure 7*b–d*). Higher magnification POM images of a 67% porous LCE film show dark areas close to the air–LC interface (figure 7*e*). This indicates the homeotropic alignment of the mesogens with the director for the polarized light oriented completely perpendicular to the surface between the crossed polarizers of the microscope. When transmitted through the material, the polarized light remains unaffected and cannot pass the second crossed polarizer resulting in the black appearance. Previous studies already showed a homeotropic anchoring at air–LC interfaces [22–24]. As the pores get wider and bigger, the thickness of the material around the pores becomes thinner. Therefore, the homeotropically aligned mesogens might not experience birefringence and look dark in the POM observation.

We assume that due to gaseous nitrogen release during polymerization, gas-release driven homeotropic alignment can take place at the LCE–air interface. Because this interface area is larger in samples with higher porosity, the volume of homeotropically aligned mesogens can be expected to be larger. A homeotropically aligned surface volume experiences more expansion in parallel to the surface in comparison to a polydomain surface when actuating under UV illumination (figure 7*f*). This relatively bigger expansion of the light-actuated homeotropically aligned mesogen volume contributes to the bending additionally.

With its lower density but larger internal surface area, a porous structure provides wider area for impact of an external stimulus. During illumination with the UV light source, the ratio of the volume in which molecular building blocks are photoisomerized to the total volume, determines the gradient of bending. Therefore, in the porous LCE with its larger apparent surface area, the same amount of light can stimulate a larger amount of azobenzene units to photoisomerization. This increase leads to a higher ratio of expanded volume to non-expanded volume and higher bending angles of porous films in comparison with non-porous films (figure 7*g*). Additionally, the presence of pores on the opposite side of the expanded volume leads to more pronounced macroscopic bending as the volume contraction at that porous surface is more favourable compared with non-porous surfaces.

The slightly higher forces on average exerted by the pore-free film may be attributed to its uniform morphology and higher amount of azobenzene units at the illuminated surface, while porous films demonstrate local shape changes around pores which might lead distribution of forces to directions other than the bending direction.

## 3. Conclusion and outlook

Different azobenzene-containing free-standing LCE films with 5, 28 and 67% porosity were produced and compared to pore-free films. The high porosity increased the photoresponse of the LCE in terms of bending angle with high significance. The photoswitching has been also demonstrated by force measurements, when the LCE films were illuminated by alternating the UV and visible light stimuli. The porous films produced forces comparable with the pore-free films.

When taking the advantages over pore-free LCEs like less material consumption, lower density and most notably the bigger framework for incorporation of other functionalities into account, the porous azobenzene LCEs can be used in particular where very small forces are of interest, for example, for micro-/nano-mechanical systems, or robotic applications and where low noise and quick mechanical output are necessary.

## 4. Material and methods

In order to prepare the LCE films, a material developed by Yamada and co-workers [25] was used, but the synthesis was modified to obtain free-standing films of different porosities: instead of a photoinitiator, a thermal initiator, [1,1-azobis (cyclohexane-carbonitrile)] was used. This compound liberated gaseous nitrogen in a thermal initiation event, causing microscale free volume in the polymerizing monomer melt and thereby generated porosity in the final LCE films (for more details, see the electronic supplementary material). Independent from volume of porosity, the maximum thicknesses for all LCE films were 60 ± 3 µm.

By varying the amount of the thermal initiator by using 1.6, 1.8 and 2 mol% in the polymerization process, three different porous LCE films with air volumes of 5, 28 and 67% were obtained, respectively. The degree of porosity could be estimated by colour threshold analysis (for more details, see the electronic supplementary material). Beside this, during our attempts with different initiator amounts, we were able to obtain pore-free film pieces at the edges of the glass cell (used for polymerization), where the gaseous nitrogen release ceased before the all monomers had been consumed.

SEM images were acquired by Hitachi S-4800 (Hitachi High-Technologies Corp., Tokyo, Japan) using 3 kV acceleration voltage. POM images of LCE films were acquired using a Zeiss Axioplan microscope in transmission mode and equipped with a Zeiss AxioCam MRc camera and the software Axio Vision (Carl Zeiss Microscopy GmbH, Jena, Germany) with aligning analyser and a polarized light filter 90° to each other.

The effective elastic modulus (*E*′) was determined by microindentation using the micro force tester Basalt-01 (TETRA GmbH, Ilmenau, Germany). The probe mounted on the calibrated steel cantilever (spring constant 203 N m^−1^) was a sapphire sphere with a radius of 0.5 mm. Indentation forces were in the range from 2.1 × 10^−3^ to 5.1 × 10^−3^ N. From the force–distance curves, the elastic moduli were obtained by fitting the Hertz model of elastic contact using Matlab R2012b (The MathWorks, Inc., Natick, MA, USA). Statistical analyses were performed using the software package SigmaPlot 12.5 (Systat Software, Inc., Richmond, CA, USA).

## Supplementary Material

Supplementary information(SI) file in PDF. The SI contains details about methods, materials and raw data used in the manuscript used.

## Data accessibility

General methods, materials used and datasets figures of this article are available in the electronic supplementary material.
