# Supplementary material for: Influence of the porosity on the photoresponse of a liquid crystal elastomer
Source: R Soc Open Sci. 2016 Apr 6;3(4):150700. doi: 10.1098/rsos.150700 (PMC4852635; doi:10.1098/rsos.150700)
Supplement: Supplementary information(SI) file in PDF. The SI contains details about methods, materials and raw data used in the manuscript used. [file rsos150700supp1.pdf]

Supplementary Information
for

Influence of Porosity on Photoresponse of Liquid Crystal Elastomer

Emre Kizilkan[1], Jan Strueben[2], Xin Jin[3], Clemens F. Schaber[1], Rainer Adelung[3]*, Anne Staubitz[2,4]*, Stanislav N. Gorb[1]*

[1] Department of Functional Morphology and Biomechanics, Zoological Institute, Kiel University, Am Botanischen Garten 1-9, 24118 Kiel, Germany
[2] Otto-Diels-Institute for Organic Chemistry, Kiel University, Otto-Hahn-Platz 4, 24118 Kiel, , Germany
[3] Institute for Materials Science, Functional Nanomaterials, Kiel University, Kaiserstr. 2, 24143 Kiel, Germany
[4] Institute for Organic and Analytical Chemistry, University of Bremen, Leobener Straße NW 2 C, 28359 Bremen, Germany

* ra@tf.uni-kiel.de; staubitz@uni-bremen.de; sgorb@zoologie.uni-kiel.de

Table of Contents

Abbreviations 3
General Methods and Materials 3
Reagents / Reactants 3
Solvents 4
Analyses 4
Synthetic and Purification Equipment 4
Porosity Calculations 5
Bending and Force Measurements 5
Polymerization 7
Glass Cell and Polymerization Methodology 7
Thermal Recovery 8
Syntheses 9
4-(9-Hydroxynonanyloxy)nitrobenzene (1) 9
4-(9-Hydroxynonanyloxy)aniline (2) 9

4-Hydroxy-4’-(9-hydroxynonanyloxy)azobenzene (3) 10
4,4’-Bis(9-hydroxynonanyloxy)azobenzene (4) 11
4-(9-Hydroxynonanyloxy)-4’-(nonanyloxy)azobenzene (5) 11
4,4’-Bis[9-(acryloyloxy)nonanyloxy]azobenzene (6) 12
4-[9-(Acryloyloxy)nonanyloxy]-4’-(nonanyloxy)azobenzene (7) 13
NMR Spectra – ^{1}H NMR Spectra followed by ^{13}C NMR Spectra 14
4-(9-Hydroxynonanyloxy)nitrobenzene (1) 14
4-(9-Hydroxynonanyloxy)aniline (2) 15
4-Hydroxy-4’-(9-hydroxynonanyloxy)azobenzene (3) 16
4,4’-Bis(9-hydroxynonanyloxy)azobenzene (4) 17
4-(9-Hydroxynonanyloxy)-4’-(nonanyloxy)azobenzene (5) 18
4,4’-Bis[9-(acryloyloxy)nonanyloxy]azobenzene (6) 19
4-[9-(Acryloyloxy)nonanyloxy]-4’-(nonanyloxy)azobenzene (7) 20
DSC-Plots 21
DSC scan rate 10 K/min, second time heating curves are shown with baseline corrections. 21
4-Hydroxy-4’-(9-hydroxynonanyloxy)azobenzene (3) 21
127 °C: Phase transition to nematic phase; 148 °C: Phase transition to isotropic melt. 21
4,4’-Bis(9-hydroxynonanyloxy)azobenzene (4) 21
64 °C: Phase transition to nematic phase; 137 °C: Phase transition to isotropic melt. 21
4-(9-Hydroxynonanyloxy)-4’-(nonanyloxy)azobenzene (5) 22
116 °C: Phase transition to isotropic melt. 22
4,4’-Bis[9-(acryloyloxy)nonanyloxy]azobenzene (6) 22
78 °C: Phase transition to nematic phase; 91 °C: Phase transition to isotropic melt. 22
4-[9-(Acryloyloxy)nonanyloxy]-4’-(nonanyloxy)azobenzene (7)3 23
69 °C: Phase transition to nematic phase; 94 °C: Phase transition to isotropic melt. 23

Abbreviations

ATR	attenuated total reflectance
at	apparent triplet (NMR)
calcd.	calculated
COSY	correlated spectroscopy
d	doublet (NMR)
DMF	*N*,*N*-dimethylformamide
DSC	dynamic scanning calorimetry
HMBC	heteronuclear multiple bond coherence
HSQC	heteronuclear single quantum coherence
IR	infrared
m	medium (concerning the intensity) (IR)
m	multiplet (NMR)
M.p.	melting point
MS	mass spectrometry
Ph	phenyl
R_f	retention factor
s	strong (concerning the intensity) (IR)
s	singlet (NMR)
t	triplet (NMR)
THF	tetrahydrofuran
w	weak (concerning the intensity) (IR)

General Methods and Materials

Reagents / Reactants

If not noted otherwise, all reagents were used as received.

**Reagent**	**Supplier**	**Purity**
Acroylchloride	Alfa Aesar Inc.	99%
Benzene-1,4-diol	Merck	99.5 %
9-Bromo-1-nonanol	Sigma-Aldrich Inc.	99%
Hydrochloricacid (37 %)	Grüssing	98%
Magnesium sulfate	Grüssing	99 %
4-Nitrophenol	Sigma-Aldrich Inc.	99 %
Phenol	Alfa Aesar Inc.	97%
Potassiumcarbonate	Grüssing	>95%
Palladium on carbon (10 %)	Alfa Aesar Inc.	98%
Sodiumnitrite	Alfa Aesar Inc.	98%
Sodiumhydroxide	Grüssing	99%
Sodiumhydride	VWR	98%
Triethylamine	Alfa Aesar Inc.	99%

Solvents

All solvents were freshly distilled, if used for purification. Where noted, solvents were dried over the specified drying agent by refluxing for several hours before distillation. Dry solvents were degassed by three freeze-pump-thaw cycles and stored in a nitrogen filled glove box over 3 Å molecular sieves.

Solvent	Supplier; drying procedure
Acetonitrile	Sigma Aldrich; Dried over phosphorus pentoxide
Chloroform	VWR; -
Cyclohexane	VWR; -
DMF	Acros Organics, extra dry, stored over 3 Å molecular sieves, flushed with nitrogen.
Ethyl acetate	BCD;-
Methanol	BCD; -
THF	Merck-Polaro, dried and degassed with an PS-MD-5 by Innovation Technology.

Analyses

^{1}H NMR spectra, and ^{13}C NMR, spectra were recorded at 300 K. ^{1}H NMR spectra were recorded on a Bruker DRX 500 (500 MHz) spectrometer or a Bruker Avance 600 spectrometer. ^{13}C NMR spectra were recorded on a Bruker DRX 500 (126 MHz) spectrometer or a Bruker Avance 600 (151 MHz) spectrometer. ^{3}C NMR spectra were referenced against the solvent residual proton signals (^{1}H) or the solvent itself (^{13}C). The exact assignment of the peaks was performed by two-dimensional NMR spectroscopy such as ^{1}H COSY, $^{1}H/^{13}C$ HSQC or $^{1}H/^{13}C$ HMBC when possible.

Ultra high resolution mass spectra were recorded on a JEOL ACCUTOF GCV JMS-T100GCVat 70 eV ionisation energy. IR spectra were recorded on a Perkin Elmer Paragon 1000 FT-IR spectrometer with a A531-G Golden-Gate-ATR-unit. Dynamic scanning calorimetry was performed on a Perkin Elmer Pyris with a temperature ramp of 10 K/minute. All melting points were recorded on an electrothermal melting point apparatus LG 1586 and are uncorrected.

Synthetic and Purification Equipment

Polymerizations were performed on a Linkam LTS-420 heating stage in between PTFE coated glass slides (see description below). Thin layer chromatography (TLC) was performed with pre-coated TLC-sheets from Macherey-Nagel GmbH & Co. KG with silica 60 and fluorescent indicator UV254. Column chromatography was performed with silica gel 60M from Macherey-Nagel GmbH & Co. KG with a size of 0.040 – 0.063 mm.

Porosity Calculations

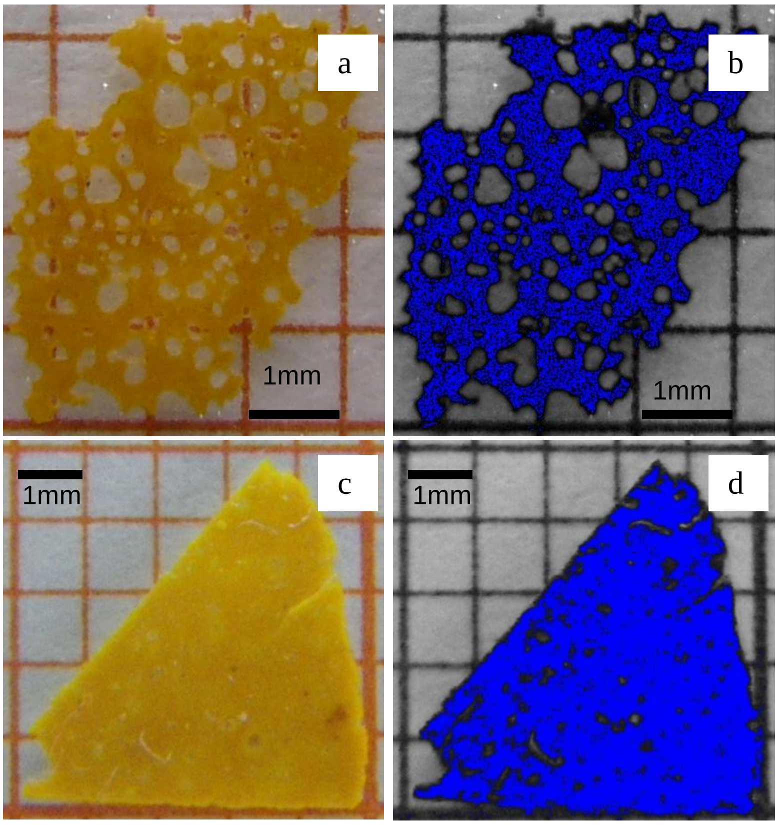

Supplementary Figure S1. Surface porosity calculation of LCE films, (a) 67% porous LCE, (b) porosity calculation of 67% porous LCE film via colour threshold, (a) 5% porous LCE, (b) porosity calculation of 5% porous LCE film via colour threshold.

The porosities of the samples were calculated with the image analysis software ImageJ 1.47v (NIH, USA). The colours of the image were separated into blue, green and red channels. The blue channel was used in the colour threshold analysis of the software, which allowed the calculation of the area of the pores of the apparent surface area of the film (Figure S1). The ratio of the calculated area to the total area was then used to estimate the porosity of the film.

Bending and Force Measurements

In the bending and force measurements, a UV LED source with λ = 365 nm (Hoenle LED UV Pen 2.0, approx. 5 W), and a homemade LED visible light source (approx. 3 W) with λ = 455nm, were used for illumination. For the photoactuation, the free standing film samples were fixed with tweezers while the lateral side of the films was illuminated with the light source.

The different light intensities were obtained by varying the distances of sample to the UV light source. The distances to the film substrate were 1.2 cm, 1.5 cm and 2 cm. At a distance of 1.2 cm, the light was in focus and light intensity was 7.5 W/cm^2 at the center of the Gaussian beam (beam diameter=~4 mm). At 1.5 cm and 2 cm, the light intensities were 80% (6 W/cm^2) and 40% (3 W/cm^2) at the center of light beam, respectively[i].The bending of the film during photoactivation was recorded with a high-speed video camera (Photron Fastcam SA1.1) The bending angle was evaluated from the displacement of the film at the point furthest from the fixed tweezer using the image analysis software ImageJ 1.47v (NIH, USA). The statistical analyses were performed by one-way ANOVA Tukey test (SigmaPlot 12.5 software, Systat Software, Inc., Richmond, CA, USA).

Figure S2 shows the measurement of forces during photoisomerisation of LCE film with 67% porosity with a size 5 x 2 x 0.1 mm. To measure forces exerted by the azobenzene LCE films during photoisomerisation, a tube-shaped glass cantilever with a diameter of 20 µm was used as force probe. The glass cantilever was marked with paint to obtain a point of reference to measure the deflection of the cantilever during the experiments. In order to calibrate the glass cantilever, a highly precise ultra micro balance (MT-SICS-UMX2, Mettler Toledo GmbH, Greifensee, Switzerland) and a motor driven micromanipulator (DC3001R with controller MS314, World Precision Instruments Inc., Sarasota, FL, USA) were used. The glass cantilever was driven by the micromanipulator onto a vertically mounted razor blade on the ultra micro balance. For each 10 µm distance driven, the weight was recorded and converted to the corresponding forces. The LCE films were brought into contact with the cantilever, so that the reference point was visible. Using a monochrome high-speed digital camera (Kodak Motion Corder Analyzer SR-Ultra, Eastman Kodak Co., San Diego, CA, USA) and an optical stereomicroscope (Leica MZ 12.5, Leica GmbH, Wetzlar, Germany), the bending of the free standing LCE films were recorded. Three LCE films with 67% porosity were used and forces were measured 40 times for each: 20 measurements under UV and 20 measurements under visible light illumination. The forces exerted by the samples on the force probe were evaluated by measuring the distance between the initial and the final positions of the reference point using ImageJ.

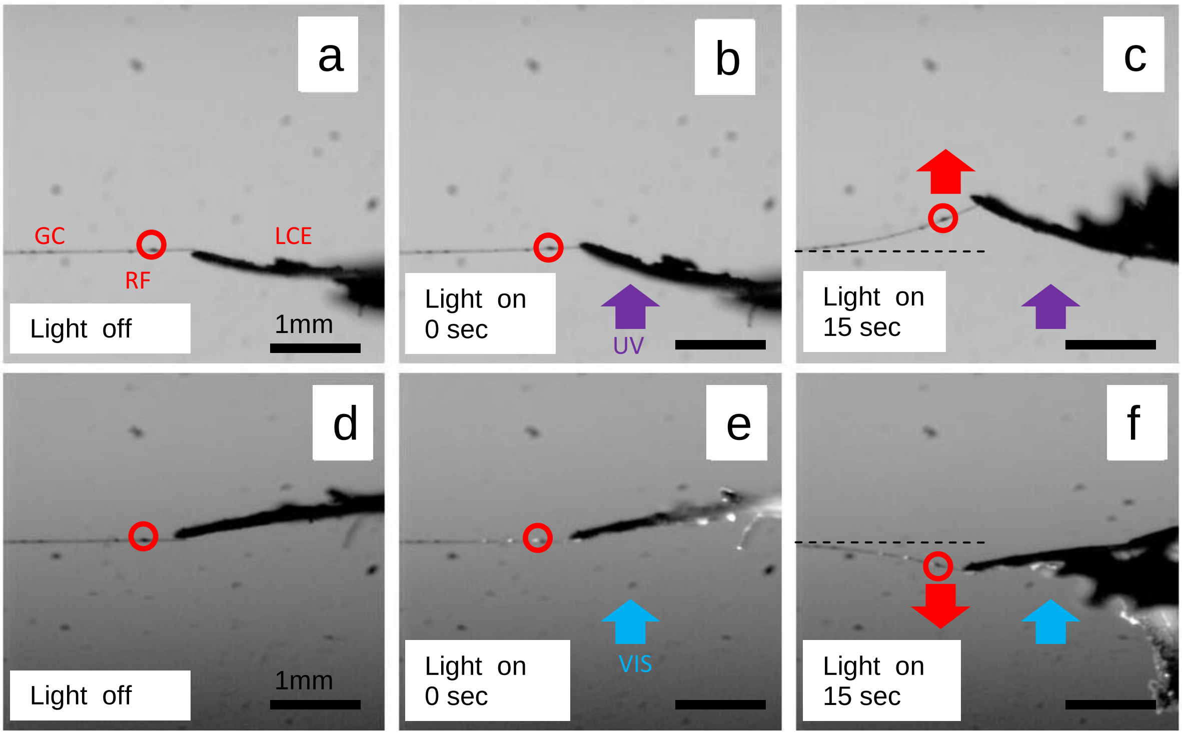

Supplementary Figure S2. Force measurements of LCE film with 67% porosity using a glass cantilever as a force probe**.** GC=glass cantilever, RF=reference point. (a-c) The film was illuminated with UV light (λ = 365 nm) was performed; (a) light source is off; (b) light source is on, 0 s; (c) light source is on, 15 s. The film bent away from the UV light illumination. This was followed by illumination with visible light (λ = 455nm) (d-f): (d) light source is off; (e) light source is on, 0 s; (f) light source is on, 15 s. After this second illumination, the film bent back again towards the source of illumination. The reference point on the glass cantilever was observed via high-speed camera and the deflections after each illumination were calculated by ImageJ.

Polymerization

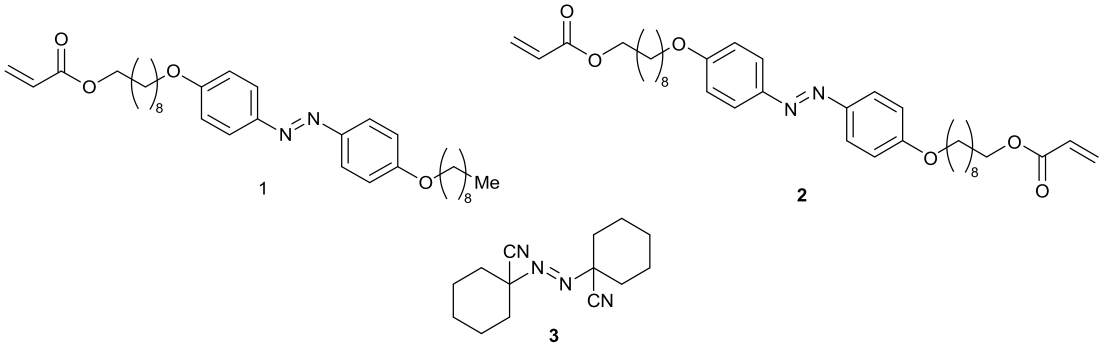

1 2 3

4(9-(acryloyloxy)nonyloxy-4’nonyloxyazobenzene (**1**, 9 eq, 18.8 mg, 31.1 µmol), 4,4’-bis[9-(acryloyloxy)nonyloxy]azobenzene (**2**, 1 eq, 150 mg, 280 µmol) and 1,1′-azobis(cyclohexanecarbonitrile) (**3**, 1.1 mg, 4.5 µmol, 1.6 mol%; 1.3 mg, 5.27 µmol, 1.8 mol% and 1.6 mg, 6.44 µmol, 2.0mol% - experiment A, B and C) were dissolved in dichloromethane. The solvent was evaporated and the solid mixture was introduced into a homemade LC cell on a heating stage (see description below). The mixture was melted at 100 °C. Then, the temperature was decreased to 91 °C over the course of 120 min. This temperature was held for 12 h (temperature stability < 0.1 °C). During the polymerization process, the gas evolution due to the decomposition of the initiator **3** produced bubbles in the polymer film. For the extraction of the polymer film from the LC cell, it was introduced in a bath of chloroform. Unreacted monomers dissolved, while the polymer film delaminated from the substrate. The resulting polymer film was extracted by soxleth extraction with chloroform for 10 h. The film was dried in a vacuum oven at 50 °C at a pressure of 10 mbar for 24 h.

Glass Cell and Polymerization Methodology

a) Fabrication of the glass cell:

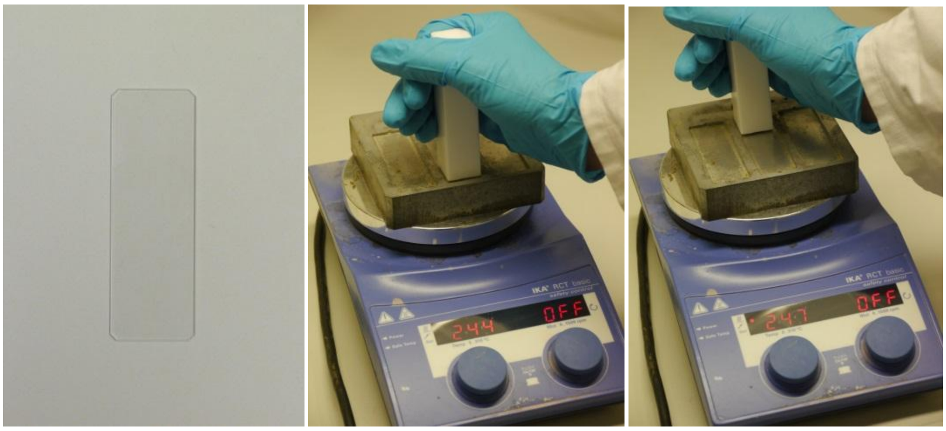

Supplementary Figure S3. A glass slide was heated on a magnetic stirrer to approx. 250 °C. A bulk PTFE stick was rubbed with a speed of approx. 7 cm / 30s over the surface of the glass slide. The method used is consistent with the previous work to produce homeotropic alignments.[ii]

b) Using the glass slides as polymerization reactors for the LCE films.

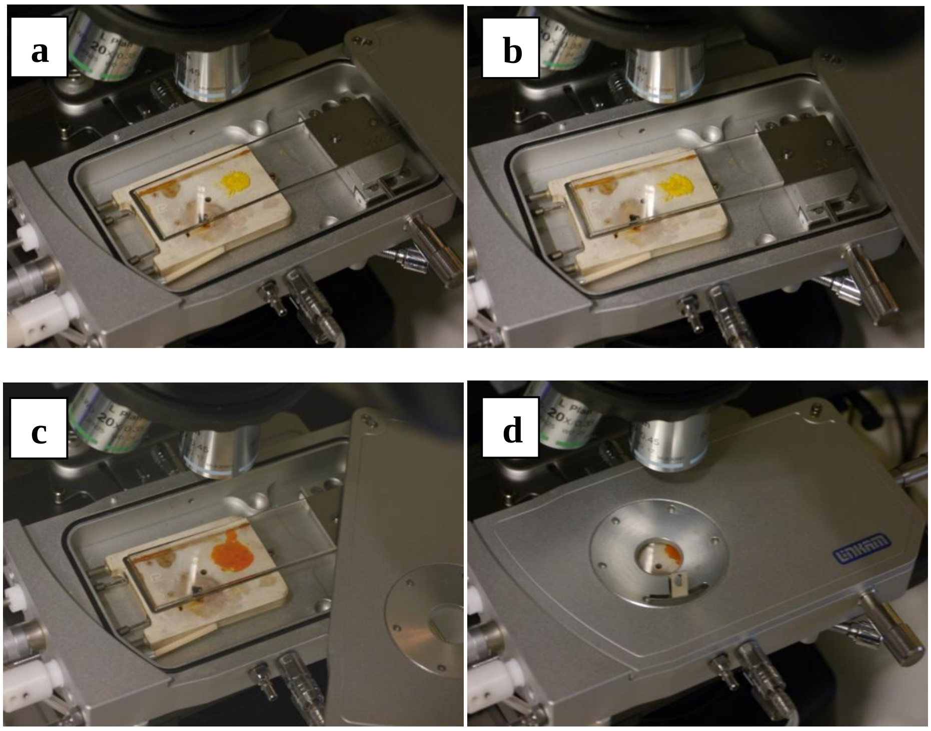

Supplementary Figure S4. A PTFE coated glass slide was placed on the heating stage. The mixture of monomers and initiator (see experimental procedure) was placed on the glass slide (a).The mixture was covered with another glass PTFE coated glass slide (b) and subsequently heated to 100 °C (at this temperature, the mixture gives an isotropic melt) (c). The stage was closed and cooled down to 94 °C over the course of 60 min. (d) The temperature was hold for 18 h while the mixture is polymerizing.

Thermal Recovery

The thermal back-isomerisation from *cis* to *trans* was completed in 24 h which is consistent with original characterisation of by Ikeda and co-workers since we used similar compounds and polymerization technique[iii]

Syntheses

The analysis of all compounds is consistent with the original characterization by Ikeda and co-workers.[iv]

4-(9-Hydroxynonanyloxy)nitrobenzene (1)

The procedure by Ikeda and coworkers was performed under Schlenk conditions in an atmosphere of dry nitrogen and modified as follows: Sodium hydride (8.96 mmol, 215 mg) was suspended in a solution of 4-nitrophenol (8.96 mmol, 1.25 g) in DMF (50,0 mL).The reaction mixture was cooled to 0 °C and a solution of 9-bromo-1-nonanol (8.96 mmol, 2.00 g) in DMF (10 mL) was added via syringe over the course of 5 min. The reaction mixture was heated at 120 °C for 90 h. After cooling to 20 °C, the residue was filtered. The solvent was evaporated in *vacuo*. The crude product was purified by column chromatography (cyclohexane : ethyl acetate, *v/v*, 2:1, R_f=0.28). A brownish white solid was obtained in a yield of 90 % (8.09 mmol, 2.28 g, Lit.:=78 %).

M. p.: 52 °C

^{1}H NMR (500 MHz, $CDCl_3$): 8.18 (d, ^3J = 9.3 Hz, 2 H, H-2), 6.93 (d, ^3J = 9.3 Hz, 2 H, H-3), 4.04 (t, ^3J = 6.5 Hz, 2 H, H-5), 3.64 (t, ^3J = 6.6 Hz, 2 H, H-13), 1.81 (m, 2 H, H-6), 1.56 (m, 2 H, H-12), 1.51-1.42 (m, 4H, H-7, H-11), 1.39-1.30 (m, 6 H, H-8, 9, 10).

^{13}C NMR (125 MHz, $CDCl_3$): 164.2 (C-4), 141.3 (C-1), 125.9 (*C-2*), 114.4 (C-3), 68.9 (C-5), 63.0 (C-13), 32.7 (C-12), 29.4 (C-9), 29.3 (C-10), 29.2 (c-8), 28.9 (C-6), 25.9 (c-7), 25.7 (C-11) ppm.[1]

IR (ATR): $\tilde{\nu}$ = 3527.6 (m), 3210 (w), 3089 (w), 2920 (s), 2879 (m), 2850 (m), 1590 (s), 1501 (s), 1473 (m), 1396 (m), 1336 (s), 1307 (m), 1253 (s), 1183 (m), 1116 (m), 1056 (m), 1031 (m), 1005 (s), 859 (s), 754 (m), 719 (m), 693 (m), 658 (s), 499 (s) cm^{-1}.

HRMS (EI): *m/z* found 281.1629; calcd. for$C_{15}H_{23}NO_4$281.1627.

4-(9-Hydroxynonanyloxy)aniline (2)

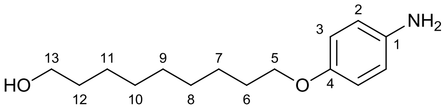

The original synthesis by Ikeda and co-workers.was modified as follows: A mixture of4-(9-hydroxynonanyloxy)nitrobenzene (**1**)(2.8 g, 10 mmol) and Pd-C (0.11 g; Pd: 10 %) was added to THF (15 mL), and the resulting suspension was stirred at 50 °C in an autoclave in a hydrogen atmosphere (150 bar) for 12 h. A precipitate formed, which was filtered and the solvent was evaporated. The crude product was dissolved in chloroform and filtered through a short column of silica with ethyl acetate as eluent. The solvent was evaporated and a yellow/brownish solid was obtained in a yield of 87 % (Lit.: 82 %).

M. p.: 87 °C

^{1}H NMR (500 MHz, $CDCl_3$): 6.73 (d, ^3J = 8.9 Hz, 2 H, 3-H), 6.62 (d, ^3J = 8.9 Hz, 2 H, 2-H), 3.86 (t, ^3J = 6.6 Hz, 2 H, H-5), 3.60 (t, ^3J = 6.7 Hz, 2 H, H-13), 1.72 (m, , 2 H, H-6), 1.53 (m, 2 H, H-12), 1.46-1.37 (m, 4H, H-7, H-11), 1.35-1.27 (m, 6 H, H-8, H-9, H-10).[2]

^{13}C NMR (125 MHz, $CDCl_3$): 153.3 (C-4), 139.7 (C-1), 116.5 (*C-2,2'*), 115.6 (C-3,3'), 68.7 (C-5), 62.8 (C-13), 32.7 (C-12), 29.5 (C-9), 29.4 (C-10), 29.3 (c-8), 29.3 (C-6), 26.0 (c-7), 25.7 (C-11) ppm.

IR (ATR): $\tilde{\nu}$ = 3337 (w), 3270 (w), 3047 (w), 2923 (s), 2853 (m), 1613 (w), 1590 (w), 1514 (s), 1475 (m), 1383 (w), 1298 (w), 1237 (s), 1104 (m), 1072 (s), 1046 (s), 1037 (m), 979 (w), 900 (m), 814 (s), 763 (s), 728 (m), 559 (m), 527 (s) cm^{-1}.

HRMS (EI): *m/z* found 251.1878; calcd. for $C_{15}H_{25}N_O$251.1885.

4-Hydroxy-4'-(9-hydroxynonanyloxy)azobenzene (3)

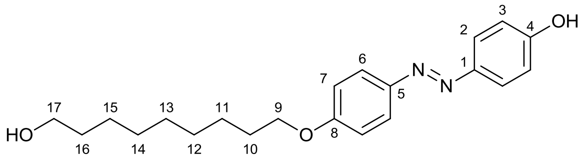

The original synthesis by Ikeda and co-workers was modified as follows: A solution of 4-(9-hydroxynonanyloxy)aniline (**2**) (8.46 g, 33.7 mmol) in hydrochloric acid (80.0 mL, 6 M) was cooled to -8 °C. A solution of sodium nitrite (2.51 g, 36.4 mmol) in water (25 ml) was cooled to 0 °C and added dropwise to the reaction mixture over the course of 15 min. A solution of phenol (3.17 g, 33.7 mmol) in an aqueous sodium hydroxide solution (100 mL, 0.75 M), cooled to - 5 °C was added dropwise over the course of 90 min. The temperature of the reaction mixture was held at - 5 °C for 30 min without stirring while the product precipitates. The precipitate was filtered and washed with water (3x 100 mL). The product was recrystallized from ethanol. A brown solid was obtained in a yield of 89 % (10.7 g) (Lit.: 59%).

DSC.: Phase Transitions at 127 °C and 148°C

^{1}H NMR (500 MHz, DMSO-*d*$_6$): 10.23 (Ar-OH), 7.78 (d, ^3J = 9.0 Hz, 2 H, H-6), 7.74 (d, ^3J = 8.9 Hz, 2 H, H-2), 7.06 (d, ^3J = 9.0 Hz, 2 H, H-7), 6.93 (d, ^3J = 8.9 Hz, 2 H, H-3), 4.03 (t, ^3J = 6.5 Hz, 2 H, H-9), 3.72 (s, 1 H, CH2-OH), 3.37 (t, *J* = 6.6 Hz, 2 H, H-17), 1.72 (m,2 H, H-10), 1.44-1.37 (m, 4 H, H-11, 16), 1.32 (m, 8 H, H-12-15) ppm.

^{13}C NMR (126 MHz, $CDCl_3$): 161.1 (C-8 or C-4)[3], 161.0 (C-8 or C-4)[3], 146.6 (C-1 or C5)[3], 145.7 (C1 or C5)[3], 124.8 (C-2), 124.4 (C-6), 116.33 (C-3), 115.3 (C-7), 68.4 (C-9), 61.2 (C-17), 33.0 (C-16), 29.5 (C-13), 29.4 (C-14), 29.2 (C-12), 29.1 (C-10), 26.0 (C-11), 25.9 (C-15) ppm.

IR (ATR): $\tilde{\nu}$ = 3304 (b), 3073 (w), 2922 (m), 2923 (s), 2852 (s), 1519 (m), 1581 (s), 1501 (m), 1470 (m), 1238 (s), 1143 (s), 1105 (m), 1070 (w), 1049 (w), 1018 (s), 970 (w), 909 (w), 843 (s), 811 (m), 772 (m), 752 (m), 723 (m), 643 (w), 548 (s), 536 (m), 502 (w) cm^{-1}.

HRMS (EI): *m/z* found 356.2091; $[M]^+$ calcd. for $C_{21}H_{28}N_2O_3$ 356.2100.

[2]The proton signals of the amine were invisible, presumably due to rapid H/D exchange.
[3]The resolution of HSQC- and HMBC-NMR spectra was insufficient to allow a precise assignment of the carbon signals because the chemical shifts were too close.

4,4'-Bis(9-hydroxynonanyloxy)azobenzene (4)

A mixture of 4-hydroxy-4'-(9-hydroxynonanyloxy)azobenzene (4.00 g, 11.0mmol) and 9-bromo-1-nonanol (2.94 g, 13.0 mmol) was dissolved in DMF (50 mL), and potassium carbonate (1.80 g, 13.0 mmol) was added to the solution in one portion. The reaction mixture was heated to reflux for 36 h. Water (700 mL) was added and the mixture was allowed to settle for 30 min without stirring. The precipitate was filtered and washed with water (3x 250 mL). The crude product was dried *in vacuo* for 24 h. The product was recrystallized from THF/ methanol (9/1, *v/v*). 2.63 g (5.28 mmol, 48 %, Lit=65 %) of a yellow solid could be obtained.

DSC.: Phase Transitions at 64 °C and 178 °C

^{1}H NMR (500 MHz, $CDCl_3$): 7.79 (d, ^{3}J = 8.7 Hz, 4 H, H-2), 6.92 (d, ^{3}J = 8.7 Hz, 4 H, H-3), 3.96 (t, ^{3}J = 6.4 Hz, 4 H, H-5), 3.67 (t, ^{3}J = 6.4 Hz, 4 H, H-13), 1.74 (m, 4 H, H-6), 1.56-1.46 (m, 8 H, CH_2), 1.44-1.36 (m, 4 H, CH_2), 1.34-1.24 (m, 14 H, CH_2) ppm.[4]

^{13}C NMR (126 MHz, $CDCl_3$): 161.4 (C-4), 146.6 (C-1), 124.3 (C-2), 114.7 (C-3), 68.3 (C-5), 63.1 (C-13), 32.8, 29.5, 29.3, 29.3, 29.3, 29.2, 26.0, 25.7 ppm.[4]

IR (ATR): $\tilde{\nu}$ = 3317 (b), 2929 (m), 2923 (m), 2850 (m), 1606 (m), 1580 (m), 1501 (m), 1472 (m), 1463 (m), 1393 (m), 1316 (m), 1246 (s), 1151 (s), 1062 (m), 1024 (s), 846 (s), 750 (m), 556 (m) cm^{-1}.

HRMS (EI): *m/z* found 498.3461; $[M]^{+}$ calcd. for$C_{30}H_{46}N_2O_4$498.3458.

4-(9-Hydroxynonanyloxy)-4'-(nonanyloxy)azobenzene (5)

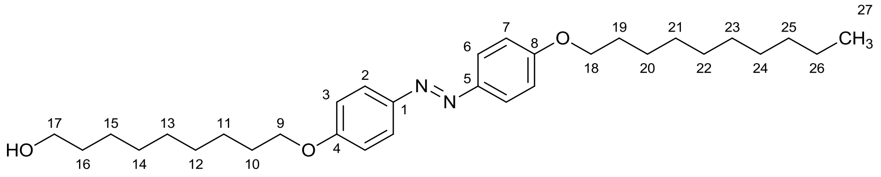

A mixture of 4-hydroxy-4'-(9-hydroxynonanyloxy)azobenzene (6.00 g, 16.8 mmol) and 9-bromo-1-nonane (3.97 mL, 19.0 mmol) were dissolved in DMF (50 mL).Potassium carbonate (2.63 g, 19.0 mmol) was added to the solution in one portion. The reaction mixture was heated to reflux for 27 h. After the reaction mixture was cooled to 20 °C, water (500 mL) was added to the mixture. The precipitate was filtered and washed with water (3 x 150 mL).The product was recrystallized from THF/ methanol (9/1, *v/v*). 4.92 g (9.91 mmol, 59%) of a yellow solid could be obtained.

M.p.: 116 °C

^{1}H NMR (600 MHz, $CDCl_3$): 7.89 (ad, ^{3}J = 8.2 Hz, 4 H, H-3, 7), 7.01 (ad, ^{3}J = 8.2 Hz, 4 H, H-2, 6), 4.06 (d, ^{3}J = 6.6 Hz, 4 H, H-9, H-18), 3.67 (at, ^{3}J = 6.6 Hz, 2 H, H-17), 1.84 (m, 4 H, H-10, H-19), 1.65-1.55 (m, 6 H), 1.54-1.56 (m, 4 H), 1.42-1.27 (m, 18 H), 0.92 (t, ^{3}J = 6.9 Hz, 3 H, H-27) ppm.[5]

---

[4]The proton and carbon signals that were not assigned could not be resolved by COSY-, HSQC- or HMBC- NMR spectra.

^{13}C NMR (150 MHz, $CDCl_3$): 161.3 (C-4, C-8), 146.8 (C-1, C-5), 124.4 (C-2, C-6), 114.7 (C-3. C-7), 68.4 (C-9), 68.3 (C-18), 63.1 (C-17), 32.8, 31.9, 29.5, 29.5, 29.4, 29.3, 29.3, 29.3, 29.2, 26.1, 25.7, 22.7 (C-26), 14.12 (C-23) ppm.[2]

IR (ATR): $\tilde{\nu}$ = 3314 (b), 3072 (w), 3053 (w), 2936 (m), 2920 (s), 2850 (s), 1603 (m), 1580 (m), 1498 (m), 1396 (m), 1246 (s), 1151 (m), 1110 (m), 1017 (s), 846 (s), 776 (m), 642 (m), 557 (s) cm^{-1}.

HRMS (EI): *m/z* found 482.3510; $[M]^+$ calcd. for $C_{30}H_{46}N_2O_3$482.3508.

4,4'-Bis[9-(acryloyloxy)nonanyloxy]azobenzene (6)

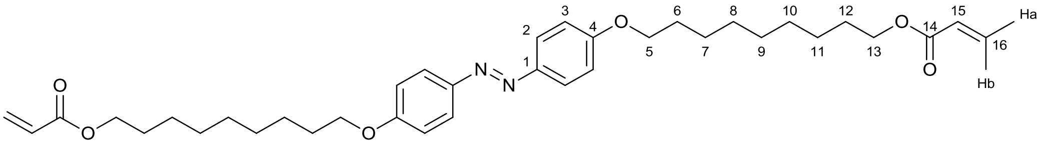

4,4'-Bis(9-hydroxynonanyloxy)azobenzene, (2.5 g, 5.0 mmol), triethylamine (4.33 mL, 31.6 mmol) and benzene-1,4-diol[6] (5.00 mg, 45.0 µmol) were dissolved in THF (500 mL). The reaction mixture was cooled to 0 °C. Acryloyl chloride (2.52 mL, 31.6 mmol) in THF (50 mL) was added dropwise over the course of 30 min. The reaction mixture was stirred for 24 h at 20 °C. It was then poured into water (500 mL), and the product was extracted with chloroform. (3 x 300 mL).The organic layer was separated and dried over magnesium sulfate. The solvent was evaporated. The crude product was purified by column chromatography (chloroform, R_f=0.56). The product was recrystallized from methanol. 1.33 g (42 %, 2.10 mmol, Lit:=25 %) of a yellow solid was obtained.

DSC.: Phase Transitions at 78 °C and 91 °C

^{1}H NMR (500 MHz, $CDCl_3$): 7.86 (d, ^3J = 8.9 Hz, 2 H, H-2), 6.98 (d, ^3J = 8.9 Hz, 2 H, H-3), 6.39 (dd, ^3J = 17.3, 1.4 Hz, 2 H, H-16b), 6.12 (6.12 (dd, ^3J = 17.3, 10.4 Hz, 2 H, H-15), 5.81 (dd, ^3J = 10.4, 1.4 Hz, 2 H, H-16a), 4.15 (at, *J* = 6.7 Hz, 4 H, H-13), 4.02 (at, J = 6.5 Hz, 4 H, H-5), 1.81 (m, 4 H, H-6), 1.67 (m, 4 H, H-12), 1.51 – 1.44 (m, 4 H, H-7), 1.42-1.31 (m, 16 H, H-8,9,10,11) ppm.

^{13}C NMR (126 MHz, $CDCl_3$): 166.3 (C-14), 161.14 (C-4), 146.95 (C-1), 130.4 (C-16), 128.6 (C-15), 124.3 (C-2), 114.7 (C-3), 68.3 (C-5), 64.7 (C-13), 29.4, 29.3, 29.2, 29.2, 28.6, 26.0, 25.9 ppm.[7]

IR (ATR): $\tilde{\nu}$ = 3070 (w), 2961 (w), 2939 (m), 2920 (s), 2853 (m), 1724 (s), 1603 (m), 1580 (m), 1498 (m), 1476 (m), 1412 (m), 1393 (m), 1320 (m), 1301 (m), 1246 (s), 1202 (s), 1018 (s), 846 (s), 556 (m) cm^{-1}.

HRMS (EI): *m/z* found 606.3671; $[M]^+$ calcd. for $C_{36}H_{50}N_2O_6$634.3669.

---

[2]The proton and carbon signals that are not assigned could not be resolved by COSY-, HSQC- or HMBC- NMR spectra.

[6] This reagent was added as an inhibitor to prevent premature polymerization of compound **6**.

[4]The proton and carbon signals that are not assigned could not be resolved by COSY-, HSQC- or HMBC- NMR spectra.

4-[9-(Acryloyloxy)nonanyloxy]-4’-(nonanyloxy)azobenzene (7)[V]

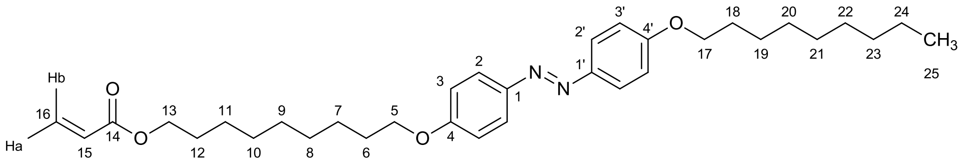

4-(9-Hydroxynonanyloxy)-4’-(nonanyloxy)azobenzene (4.50 g, 9.66 mmol), triethylamine (4.17 mL, 30.5mmol) and benzene-1,4-diol (2.1 mg, 18.9 µmol) were dissolved in THF (500 mL). The reaction mixture was cooled to 0 °C. Acryloyl chloride (2.43 mL, 30.5 mmol) in THF (50 mL) was added dropwise over the course of 30 min. The reaction mixture stirred at 20 °C for 24 h. The reaction mixture was poured into water (500 mL), and the product was extracted with chloroform (3 x 300 mL). The organic layer was separated and dried over magnesium sulfate. The solvent was evaporated. The crude product was purified by column chromatography (chloroform, R_f=0.85). The product was recrystallized from methanol. 2.74 g (49 %, 2.10 mmol) of a yellow solid were obtained.

DSC.: Phase Transitions at 69 and 94 °C

^{1}H NMR (500 MHz, $CDCl_3$): 7.86 (d, ^3J = 8.9 Hz, 2 H, H-2), 6.98 (d, ^3J = 8.9 Hz, 2 H, H-3), 6.40 (dd, J = 17.3, 1.5 Hz, 1 H, H-16b), 6.12 (6.12 (dd, ^3J = 17.3, 10.4 Hz, 1 H, H-15), 5.81 (dd, J = 10.4, 1.5 Hz, 1 H, H-16a), 4.15 (t, ^3J = 6.7 Hz, 2 H, H-13), 4.02 (at, ^3J = 6.5 Hz, 4 H, H-5, 17), 1.81 (m, 4 H, H-6, 18), 1.68 (m, 2 H, H-12), 1.48 (m, 4 H, H-7,19), 1.42-1.22 (m, 18 H, H-8-11; H-20-23), 0.89 (t, ^3J = 7.0 Hz, 3 H-25) ppm.

^{13}C NMR (126 MHz, $CDCl_3$): 166.3 (C-14), 161.2 (C-4,4‘), 147.0 (C-1,1‘), 130.4 (C-16), 128.7 (C-15), 124.3 (C-2), 114.65 (C-3), 68.3 (C-5), 68.3 (C-17), 64.7 (C-13), 31.9, 29.5, 29.4, 29.3, 29.2, 29.2, 29.2, 28.6, 26.0, 26.0, 22.7, 14.09 (C-25) ppm.[8]

IR (ATR): $\tilde{\nu}$ = 3104 (w), 3079 (w),3044 (w),2939 (m), 2923 (m), 2853 (m), 2853 (m), 1727 (m), 1711 (s), 1600 (m), 1581 (m), 1472 (m), 1476 (m), 1412 (m), 1320 (m), 1301 (m), 1240 (s), 1192 (s), 1107 (m), 1021 (s), 992 (m), 811 (m), 779 (m), 725 (m), 642 (m), 550 (m) cm^{-1}.

HRMS (EI): *m/z* found 536.3612; $[M]^+$ calcd. for $C_{36}H_{54}N_2O_4$536.3614

---

[8]The proton and carbon signals that are not assigned could not be resolved by COSY-, HSQC- or HMBC- NMR spectra.

NMR Spectra – ^{1}H NMR Spectra followed by ^{13}C NMR Spectra

4-(9-Hydroxynonanyloxy)nitrobenzene (1)

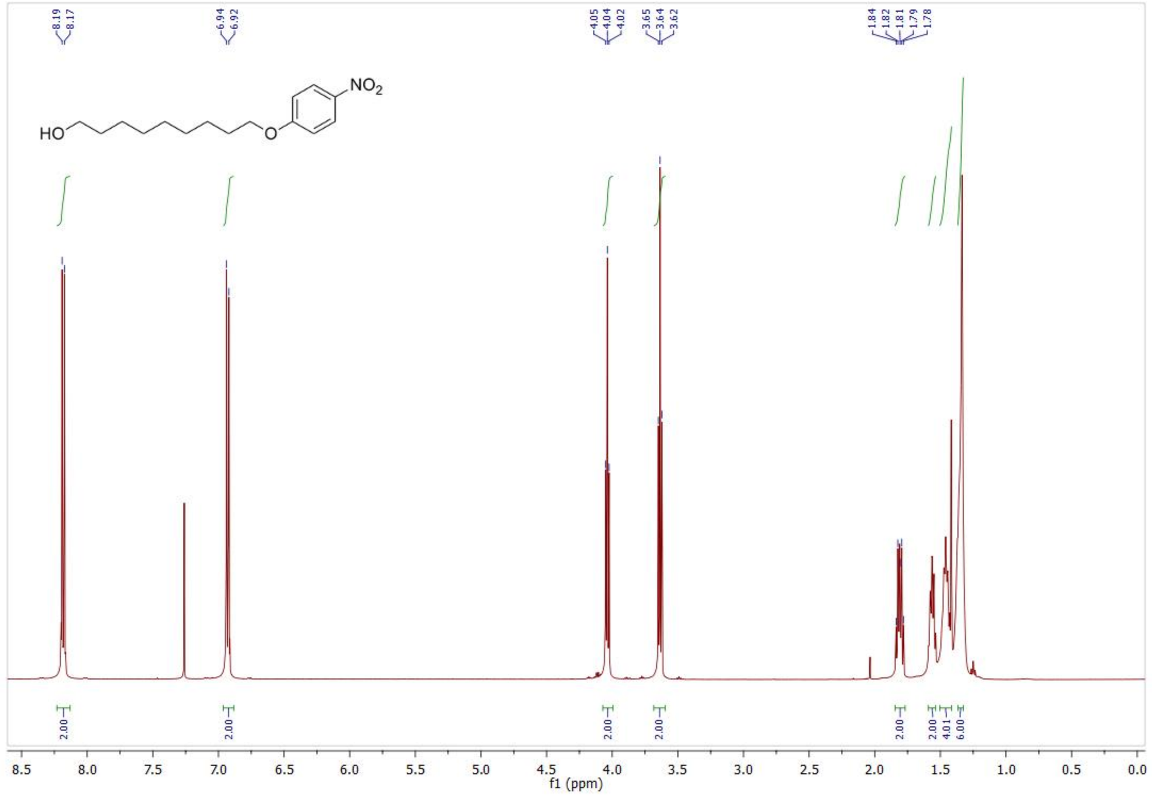

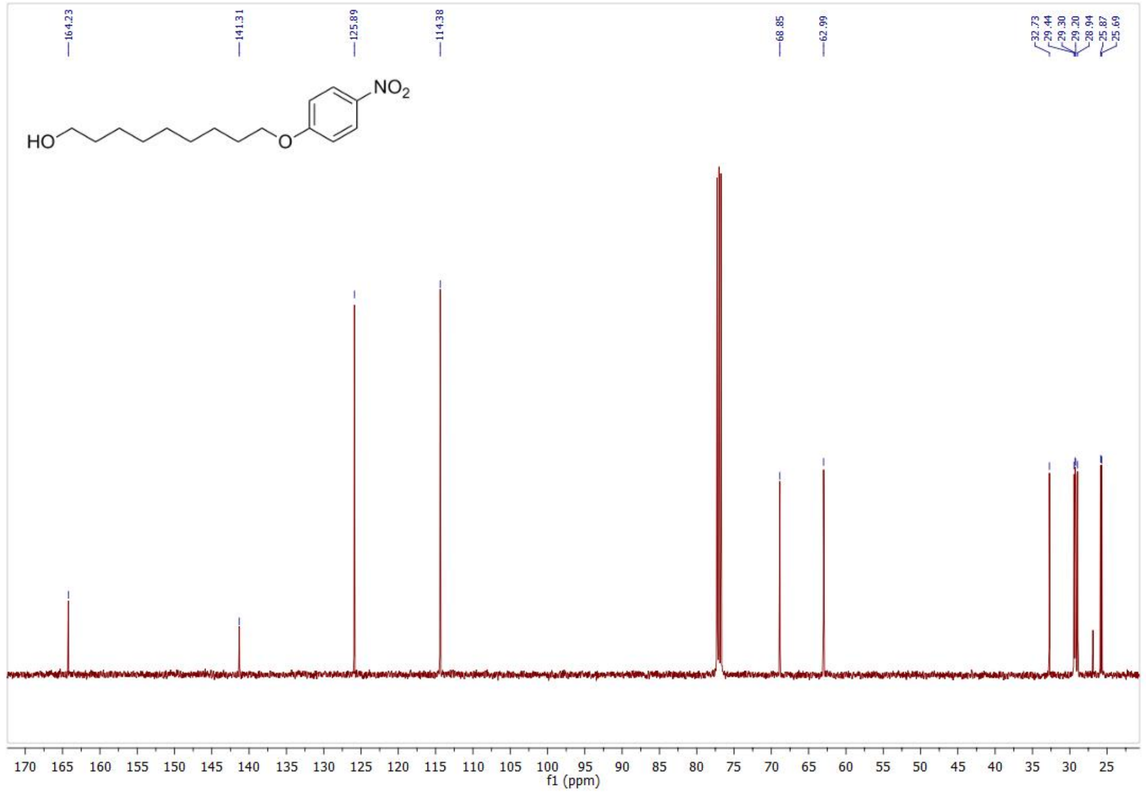

4-(9-Hydroxynonanyloxy)aniline (2)

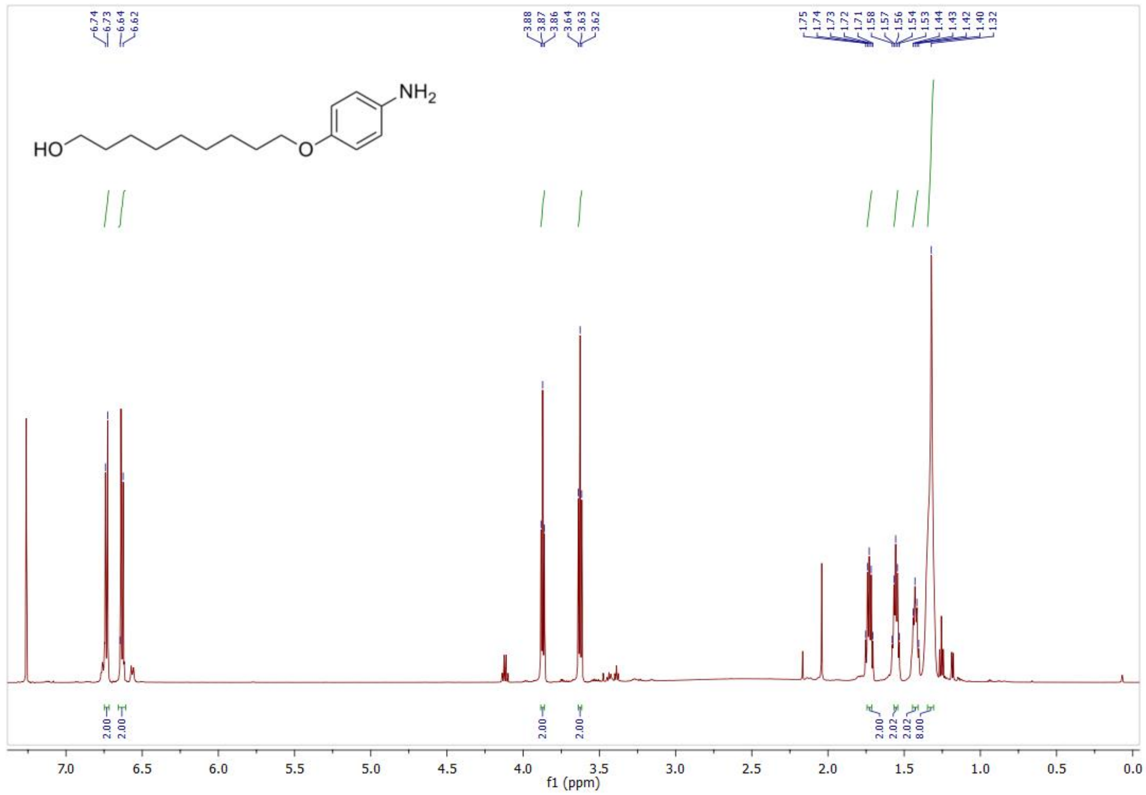

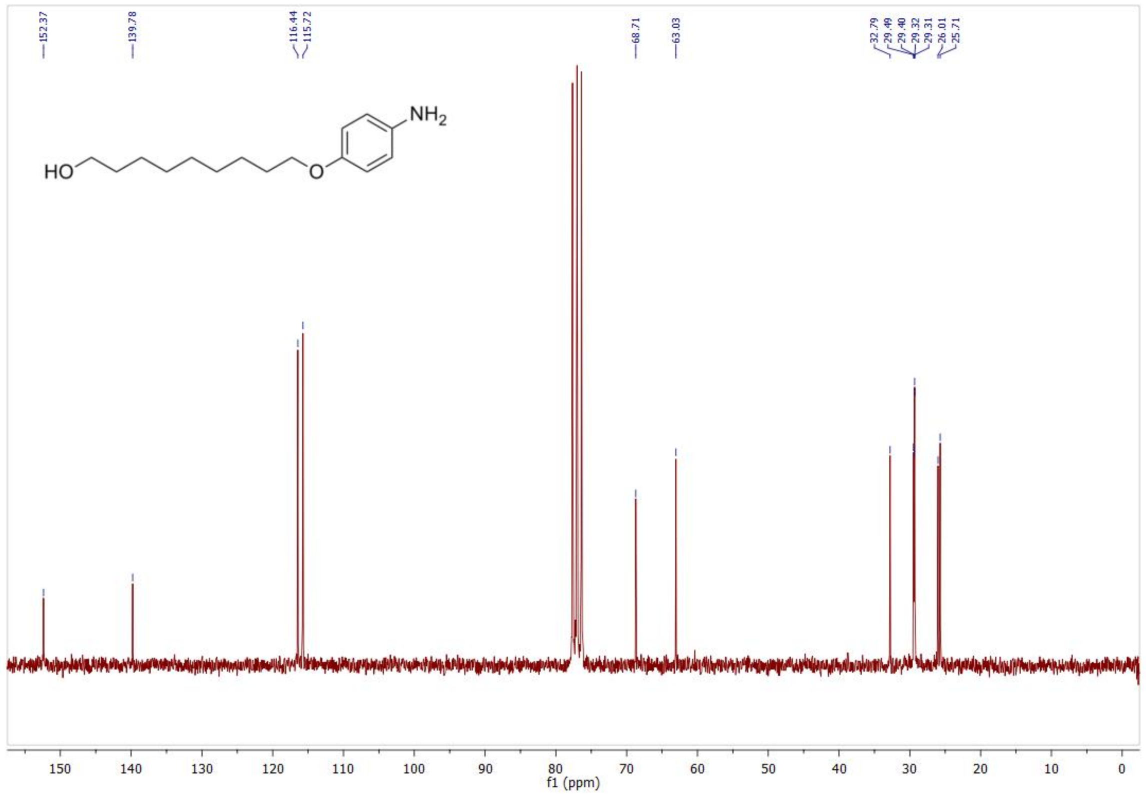

4-Hydroxy-4’-(9-hydroxynonanyloxy)azobenzene (3)

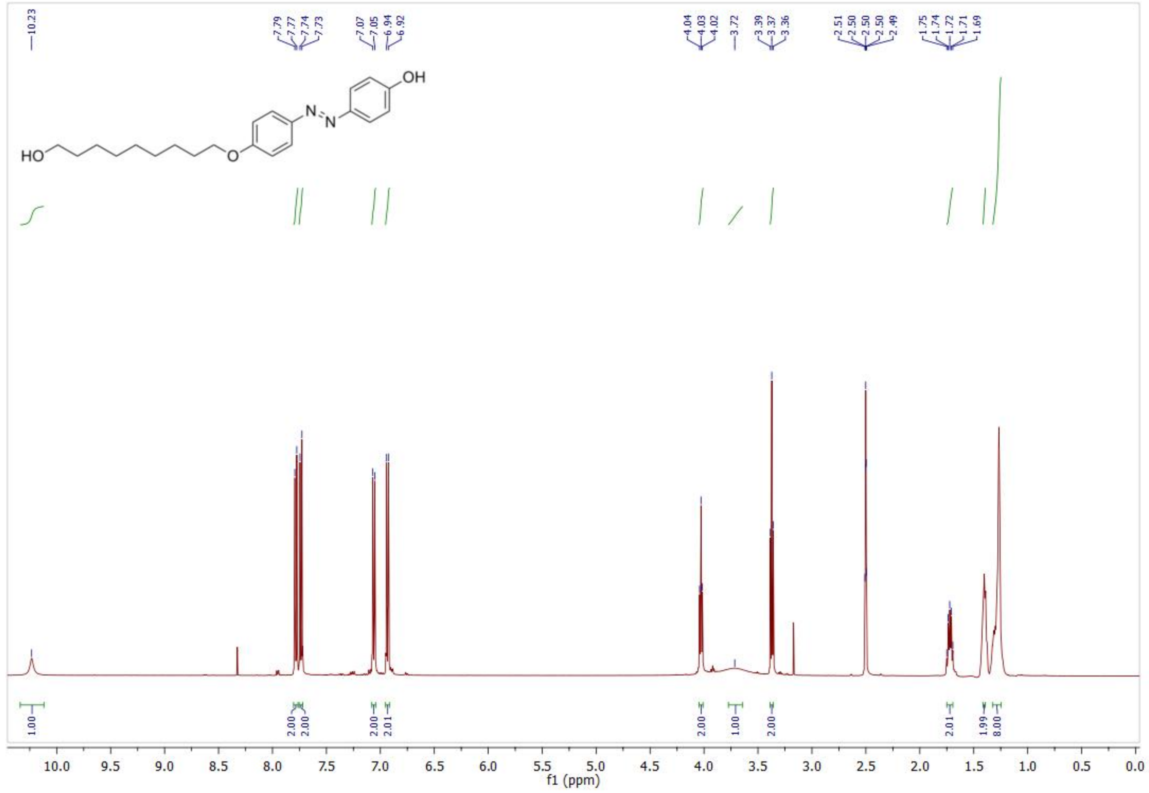

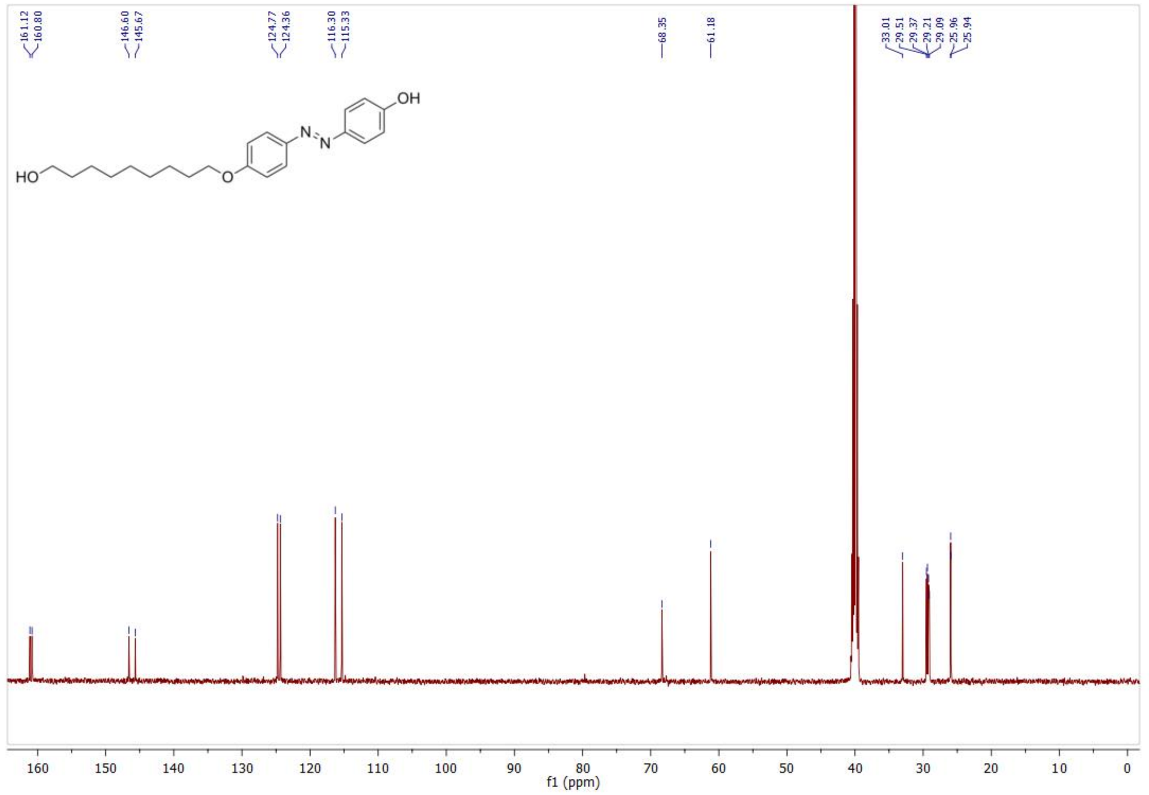

4,4’-Bis(9-hydroxynonanyloxy)azobenzene (4)

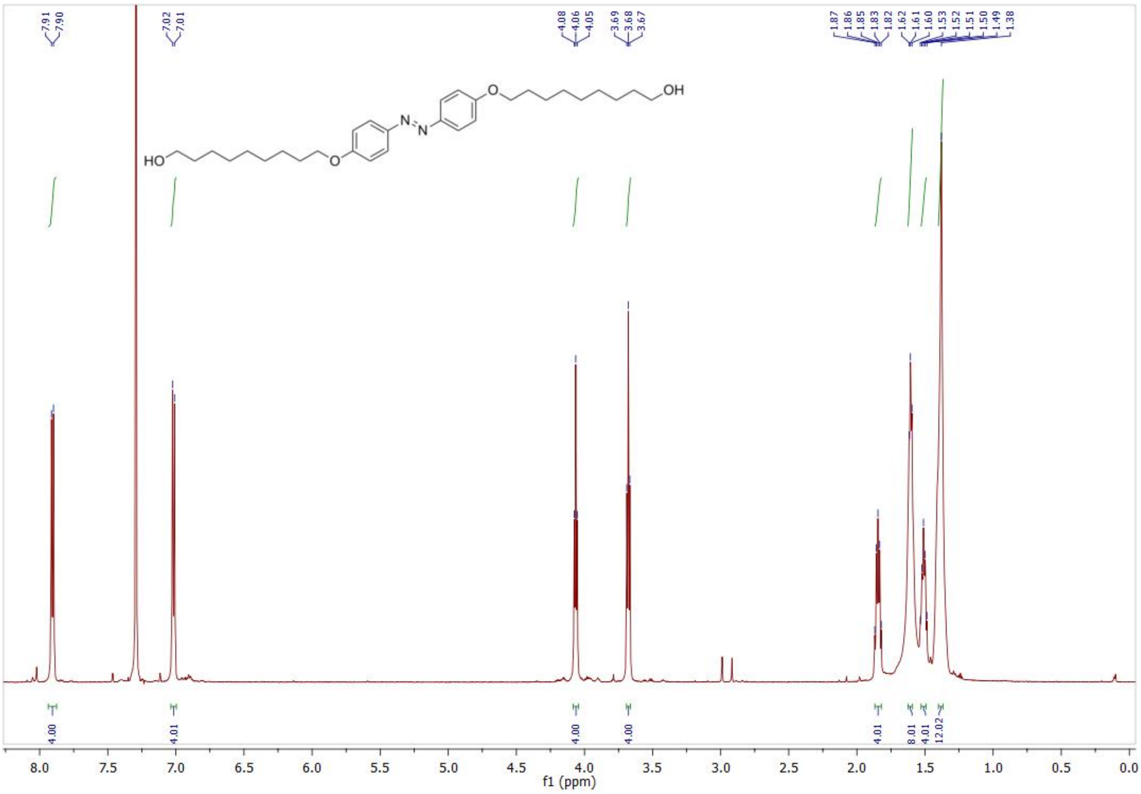

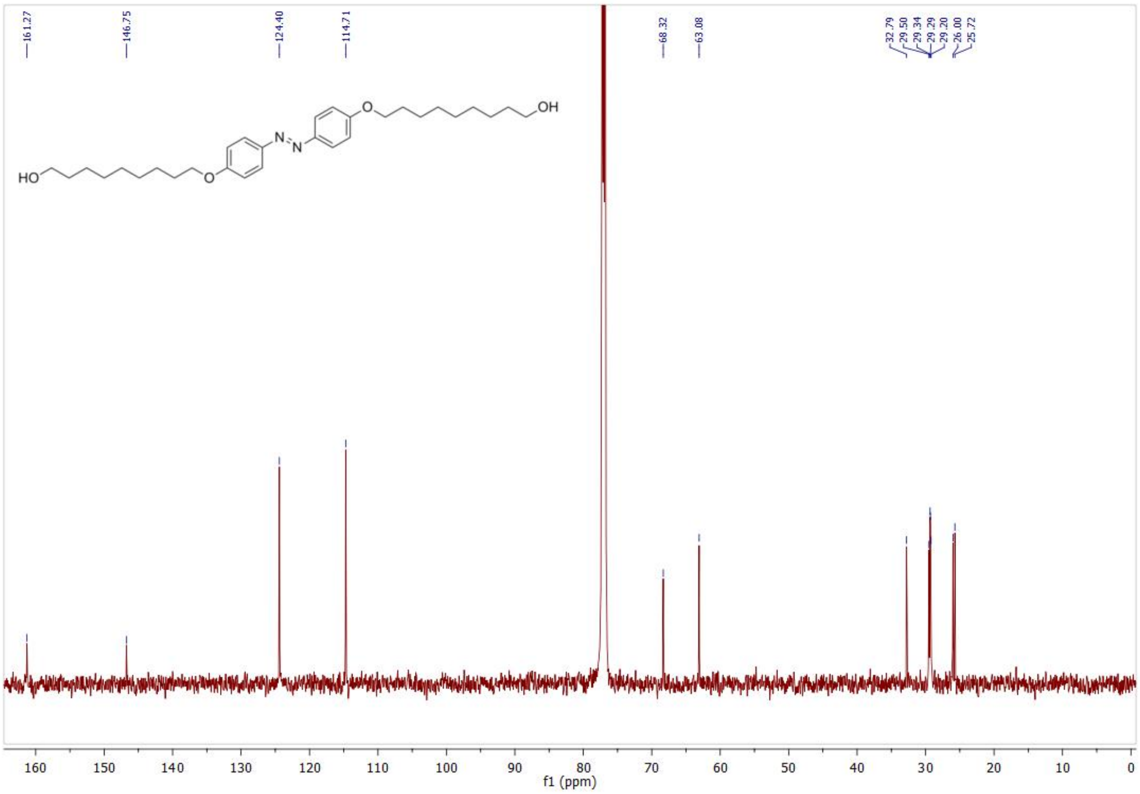

4-(9-Hydroxynonanyloxy)-4'-(nonanyloxy)azobenzene (5)

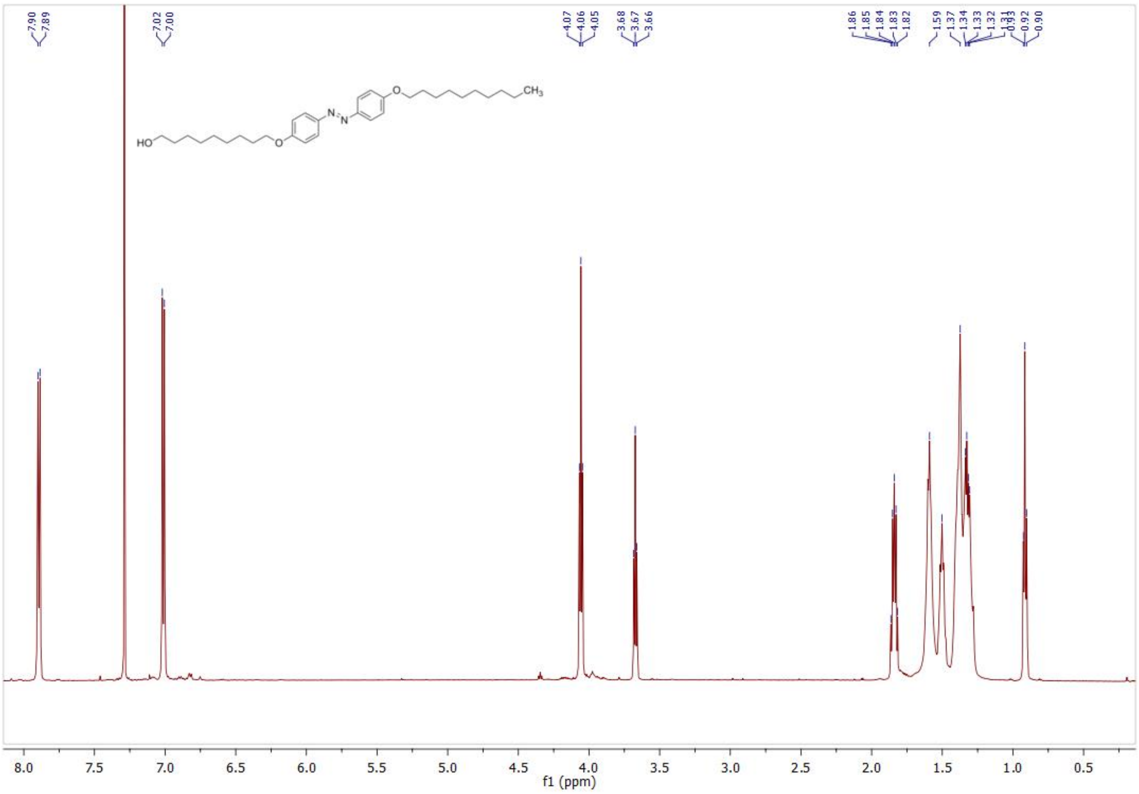

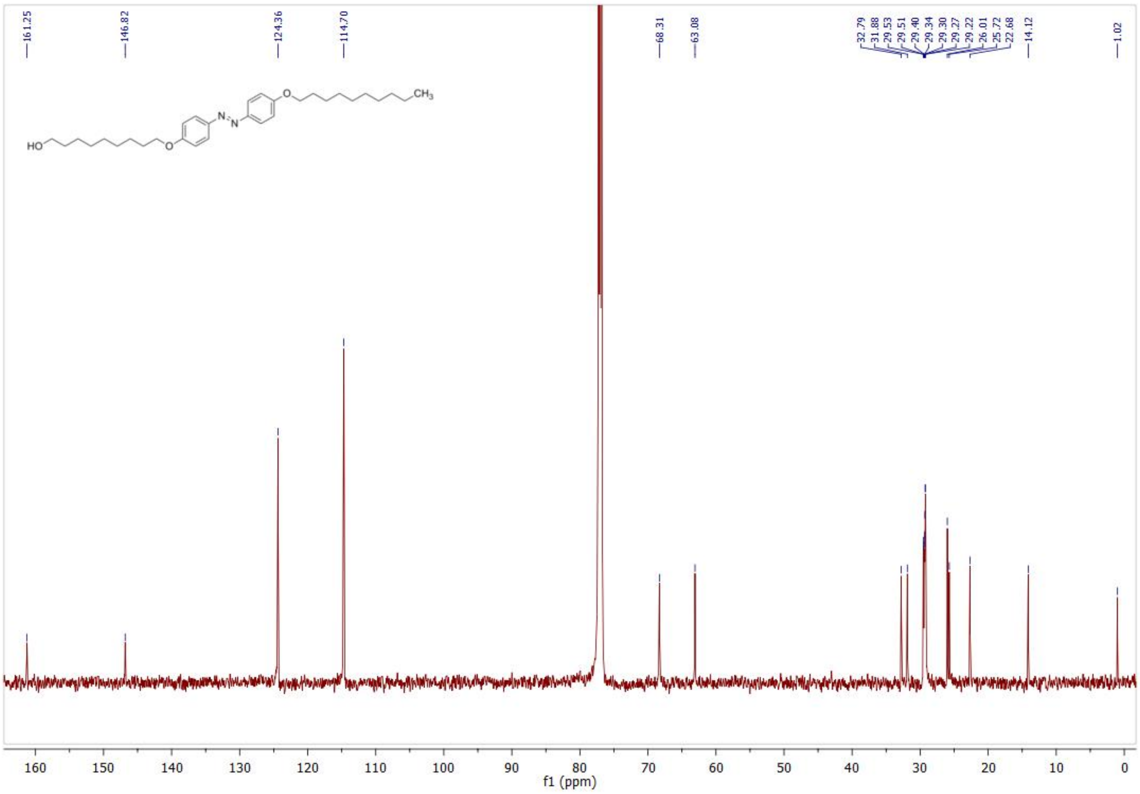

4,4'-Bis[9-(acryloyloxy)nonanyloxy]azobenzene (6)

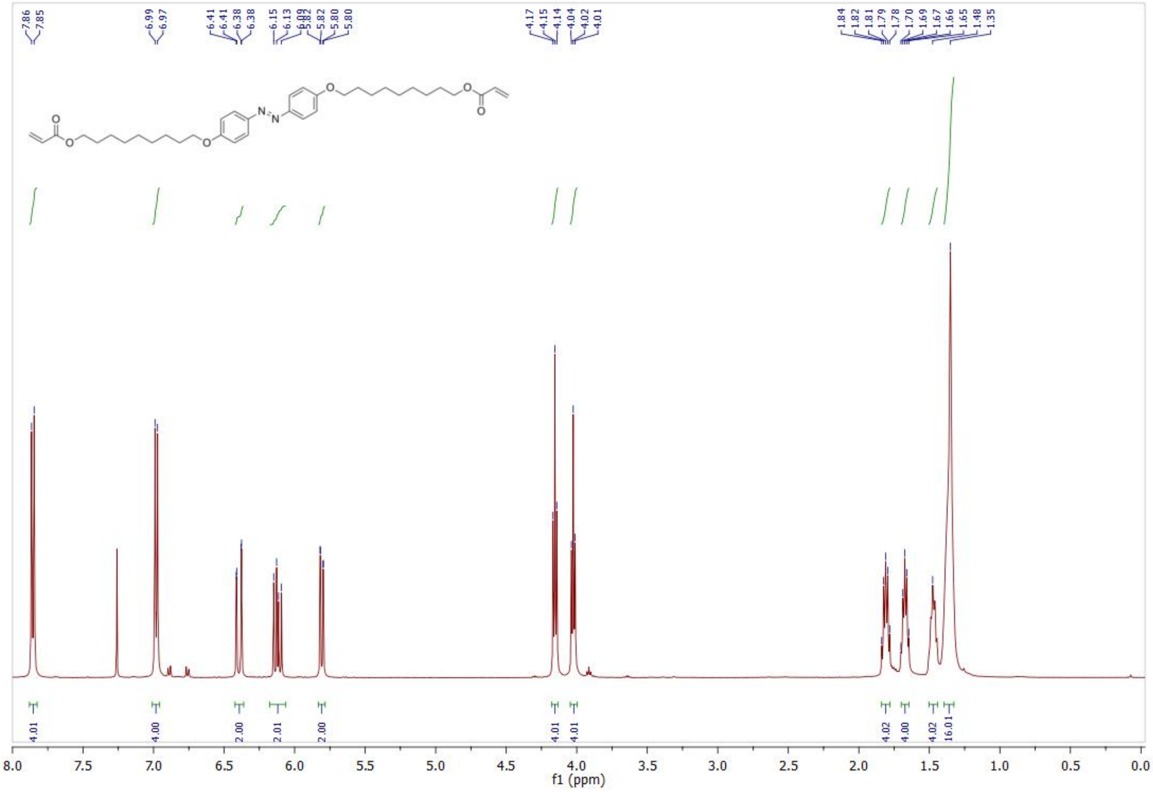

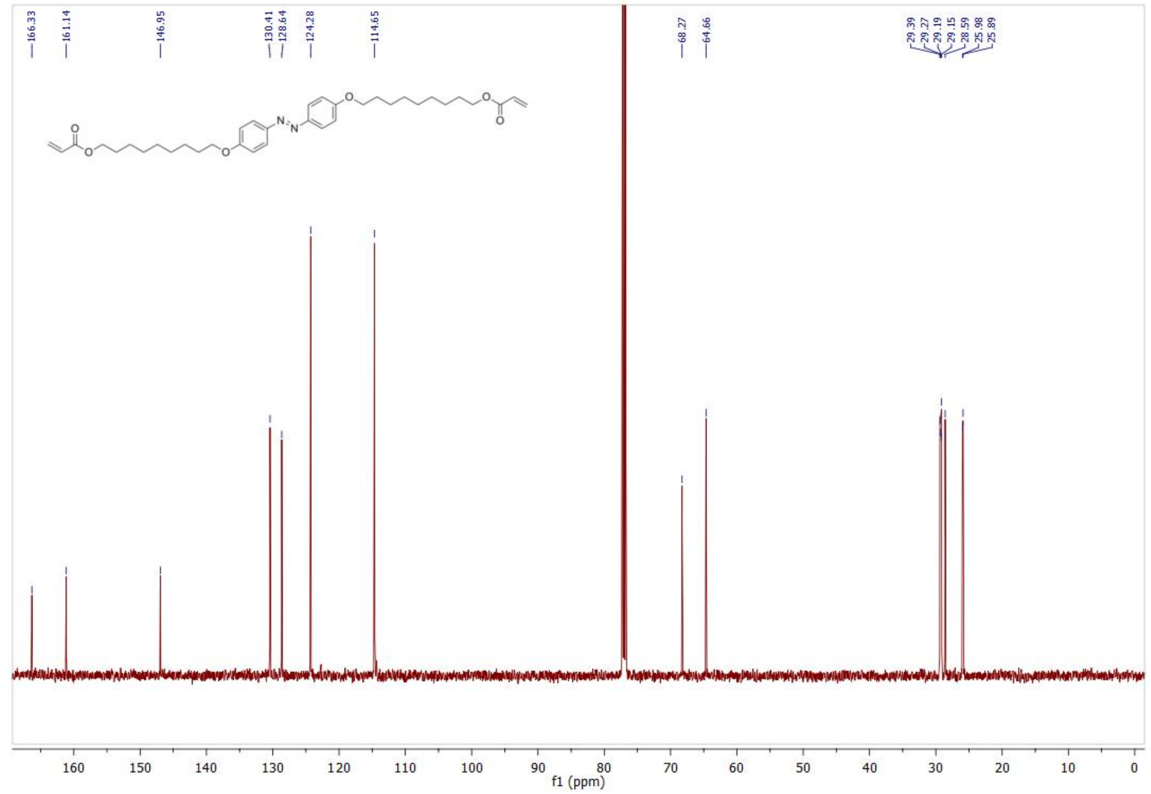

4-[9-(Acryloyloxy)nonanyloxy]-4’-(nonanyloxy)azobenzene (7)

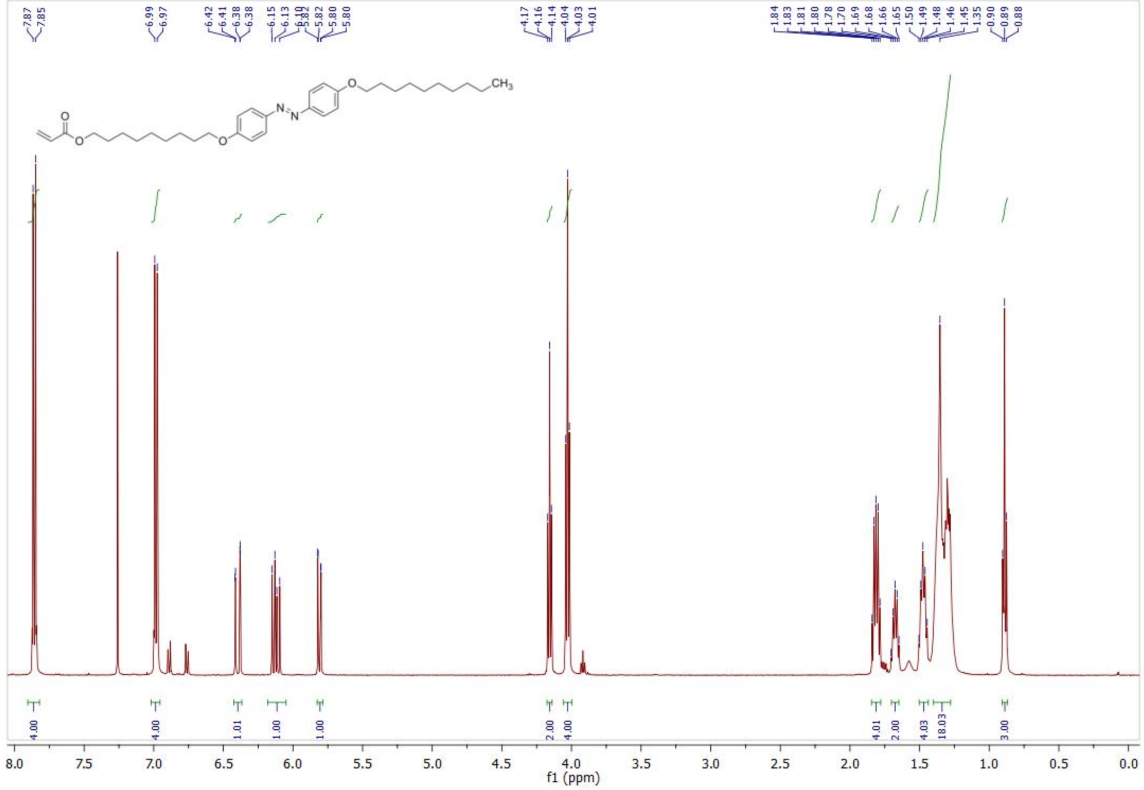

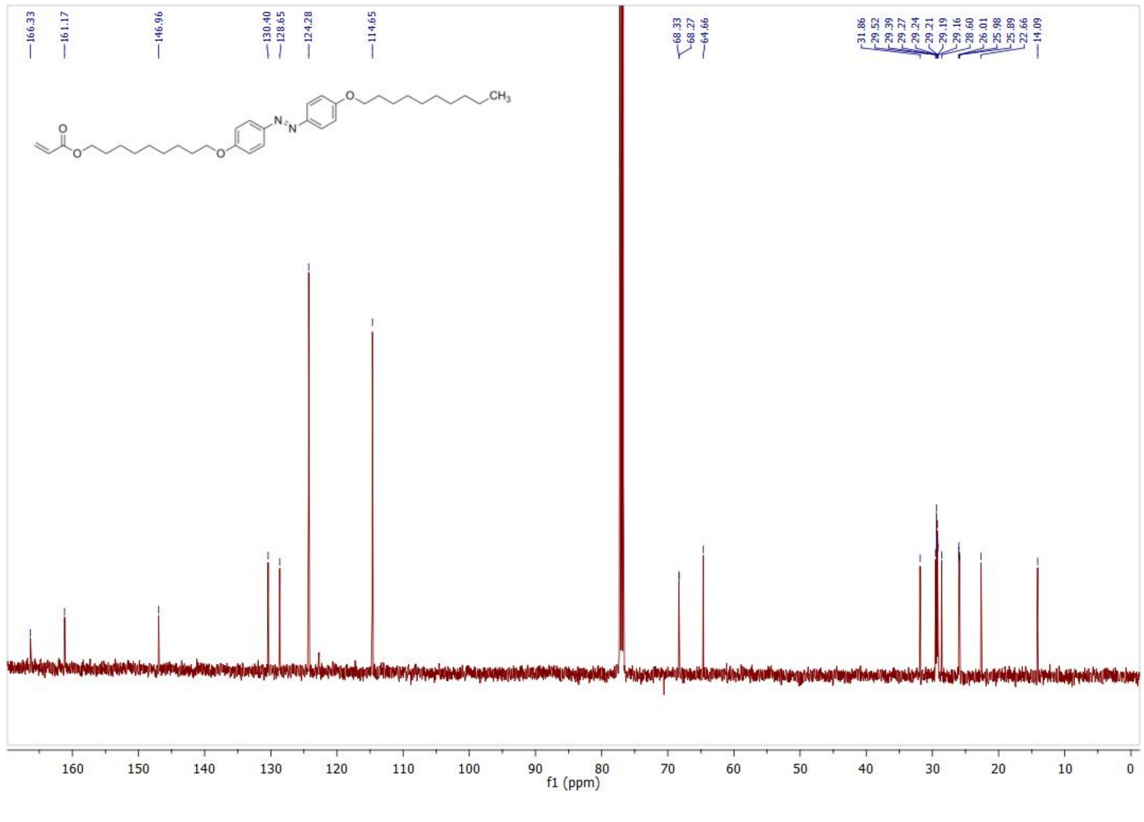

DSC-Plots

DSC scan rate 10 K/min, second time heating curves are shown with baseline corrections.

4-Hydroxy-4’-(9-hydroxynonanyloxy)azobenzene (3)

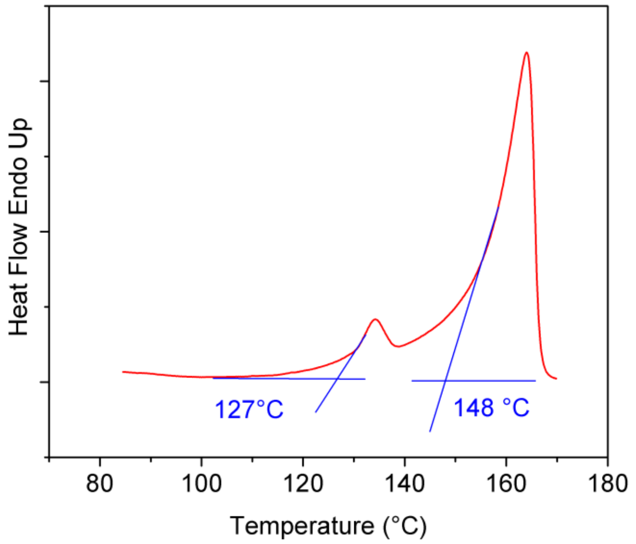

127 °C: Phase transition to nematic phase; 148 °C: Phase transition to isotropic melt.

4,4’-Bis(9-hydroxynonanyloxy)azobenzene (4)

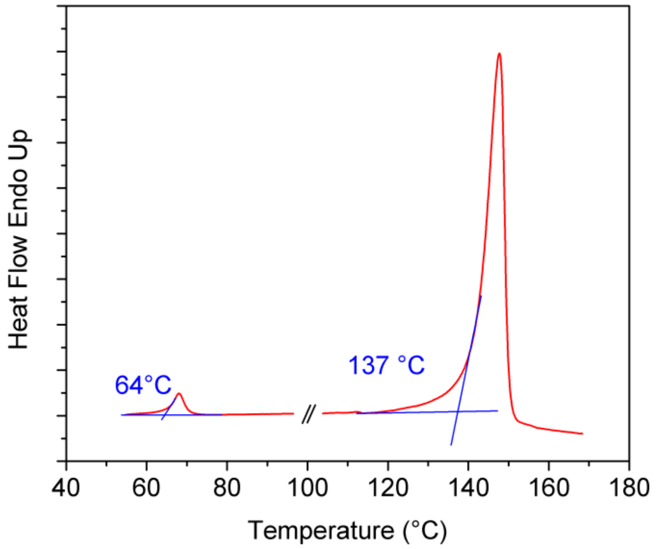

64 °C: Phase transition to nematic phase; 137 °C: Phase transition to isotropic melt.

4-(9-Hydroxynonanyloxy)-4’-(nonanyloxy)azobenzene (5)

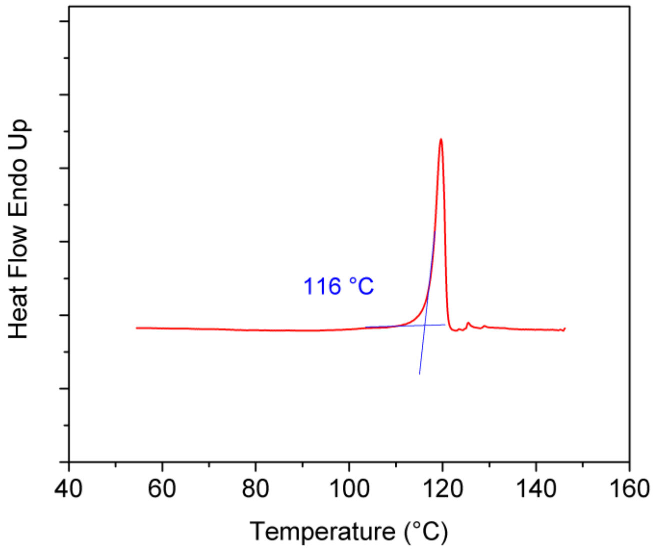

116 °C: Phase transition to isotropic melt.

4,4’-Bis[9-(acryloyloxy)nonanyloxy]azobenzene (6)

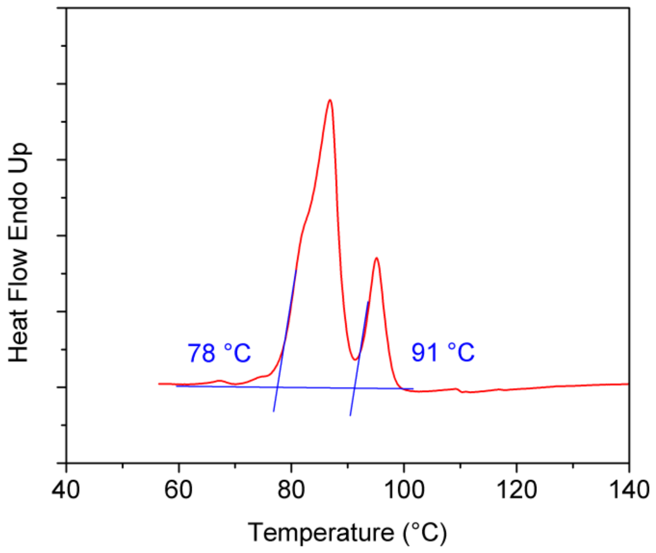

78 °C: Phase transition to nematic phase; 91 °C: Phase transition to isotropic melt.

4-[9-(Acryloyloxy)nonanyloxy]-4’-(nonanyloxy)azobenzene (7)3

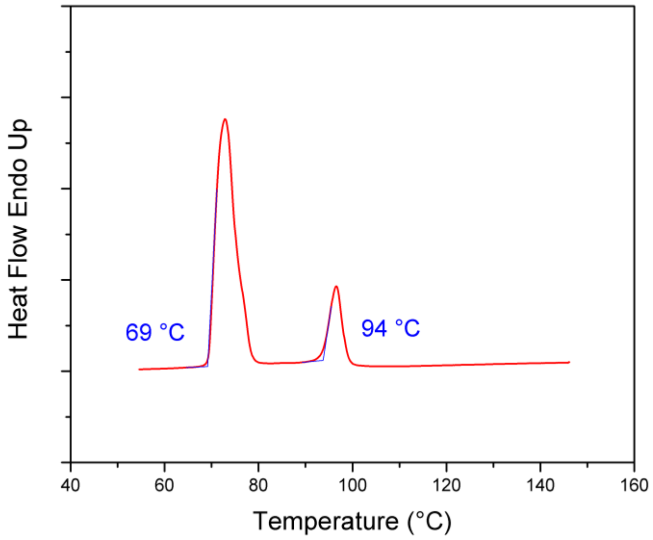

69 °C: Phase transition to nematic phase; 94 °C: Phase transition to isotropic melt.

---

[i] Dr. Hönle AG, LED Power Pen Betriebsanleitung, Germany, October 2013, page 27.

[ii] R. I. Gearba, D. V. Anokhin, A. I. Bondar, W. Bras, M. Jahr, M. Lehmann and D. A. Ivanov, *Advanced Materials*, 2007, **19**, 815-820.

[iii] Ikeda, Tomiki, and Osamu Tsutsumi. Science, 1995, 268, 1873-1875.

[iv] M. Yamada, M. Kondo, J. Mamiya, Y. Yu, M. Kinoshita, C. J. Barrett, T. Ikeda, *Angew. Chem.* 2008, **120**, 5064–5066.

[v] M. Schadt, V. Chigrinov, H. S. Kwok, S. Nose, N. Sayaka, Y. Nagashima, I. Nishiyama, H. Takatsu, *Polymer for use in liquid-crystal alignment layer*, WO2011122598 (A1), Oct. 6, 2011.
